# Impact of pantoprazole on absorption and disposition of hydroxychloroquine, a drug used in Corona Virus Disease-19 (Covid-19): A structured summary of a study protocol for a randomised controlled trial

**DOI:** 10.1186/s13063-020-04476-y

**Published:** 2020-06-29

**Authors:** Felicitas Stoll, Antje Blank, Gerd Mikus, David Czock, Kathrin I. Foerster, Simon Hermann, Katja Häußler, Amin Muhareb, Simone Hummler, Johanna Weiss, Jürgen Burhenne, Walter E. Haefeli

**Affiliations:** Heidelberg University Hospital, Heidelberg, Baden-Württemberg Germany

**Keywords:** COVID-19, Randomised controlled trial, Trial protocol, Hydroxychloroquine, Pantoprazole, Absorption, Drug-drug interaction, Microdosing, Midazolam, Yohimbine

## Abstract

**Objectives:**

Primary objective: Evaluation of the effect of the proton pump inhibitor (PPI) pantoprazole on the absorption of hydroxychloroquine (HCQ).

Secondary objectives:

• Evaluation of the relationship between HCQ concentrations in whole blood, plasma and intracellular concentrations in target cells - peripheral blood mononuclear cells (PBMCs).

• Evaluation of HCQ as a potential perpetrator in drug-drug interactions at the level of cytochrome P450 (CYP) 3A4 and CYP2D6 (major drug metabolizing enzymes).

**Trial design:**

Single centre, open-label, parallel group, two-arm, phase I drug-drug interaction trial.

**Participants:**

Healthy volunteers (18-60 years old) are treated in the Clinical Pharmacological Trial Center of Heidelberg University Hospital, Germany.

**Intervention and comparator:**

• Participants are randomized in a group to either receive a nine-day course of pantoprazole, or to a control group without pantoprazole. All participants receive a single dose of HCQ 400 mg.

• Additionally, CYP3A4 and CYP2D6 phenotyping with microdosed probe drugs is performed using midazolam and yohimbine as enzyme activity markers, respectively.

**Main outcomes:**

Primary endpoint:

Area under the curve (AUC)_0-72 h_ and maximum concentration (C_max_) of a single oral dose of 400 mg HCQ with and without pantoprazole (changes in these two values describe relevant aspects of exposure to HCQ with and without administration of pantoprazole).

Secondary endpoints:

• AUC_2-4 h_, AUC_0-6 h_ and C_max_ of midazolam and yohimbine.

• Correlation of HCQ concentrations in whole blood with concentrations in plasma and peripheral blood mononuclear cells (PBMC).

**Randomisation:**

Participants are assigned to treatment groups by using a randomisation list (1:1, block size = 4) and consecutive enrolment.

**Blinding (masking):**

The trial is an open-label trial, participants and investigators are not blinded.

**Numbers to be randomised (sample size):**

A total number of 24 participants (12 per group) are planned to be randomised.

**Trial Status:**

Protocol version 2.1 dated 24/04/2020, first patient first visit. April 30th, 2020, recruitment ongoing, anticipated end of study June 30th, 2020.

**Trial registration:**

EudraCT Number: 2020-001470-30, registered on 31 March 2020 German Clinical trials register number / International Clinical Trials Registry Platform: DRKS00021573, registered on 27 April 2020

**Full protocol:**

The full trial protocol is attached as an additional file, accessible from the Trials website (Additional file 1). In the interest in expediting dissemination of this material, the familiar formatting has been eliminated; this Letter serves as a summary of the key elements of the full trial protocol. The trial protocol has been reported in accordance with the Standard Protocol Items: Recommendations for Clinical Interventional Trials (SPIRIT) guidelines (Additional file 2).

## Supplementary information

**Additional file 1.** Full Study Protocol.

**Additional file 2.** SPIRIT 2013 Checklist: Recommended items to address in a clinical trial protocol and related documents.

## Data Availability

Data will be available from the author on reasonable request.

