# Supplementary material for: Impact of pantoprazole on absorption and disposition of hydroxychloroquine, a drug used in Corona Virus Disease-19 (Covid-19): A structured summary of a study protocol for a randomised controlled trial
Source: Trials. 2020 Jun 29;21:584. doi: 10.1186/s13063-020-04476-y (PMC7322698; doi:10.1186/s13063-020-04476-y)
Supplement: Supplementary file 1 — Additional file 1. Full Study Protocol. [file 13063_2020_4476_MOESM1_ESM.docx]

| *CLINICAL TRIAL PROTOCOL*  ***Impact of pantoprazole on absorption and disposition of hydroxychloroquine, a drug used in Corona Virus Disease-19 (Covid-19)***  *Deutscher Titel:*  *Einfluss von Pantoprazol auf Absorption und Disposition von Hydroxychloroquin als Wirkstoff für Corona-Virus-Disease-19 (Covid-19)*  *EudraCT Number:* ***2020-001470-30***  *Protocol code: K724* |
| --- |

| **SPONSOR** | Ruprecht-Karls-University Heidelberg, Medical Faculty  represented in law by Heidelberg University Hospital  and its Commercial Managing Director Katrin Erk Im Neuenheimer Feld 672 69120 Heidelberg |
| --- | --- |
| **PRINCIPAL INVESTIGATOR** | Prof. Dr. med. Walter E. Haefeli  Heidelberg University Hospital Department of Clinical Pharmacology and Pharmacoepidemiology Im Neuenheimer Feld 410 69120 Heidelberg Tel.: +49 (0) 6221 / 56- 8740 Fax: +49 (0) 6221 / 56- 4642 Email: Walter.Emil.Haefeli@med.uni-heidelberg.de |
| **DEPUTY INVESTIGATOR** | Dr. med. Antje Blank  Heidelberg University Hospital Department of Clinical Pharmacology and Pharmacoepidemiology Im Neuenheimer Feld 410 69120 Heidelberg Tel.: +49 (0) 6221 / 56- 39537 Fax: +49 (0) 6221 / 56- 8523 Email: antje.Blank@med.uni-heidelberg.de |
| **DEPUTY INVESTIGATOR** | Dr. med. Dipl. Vw. Felicitas Stoll  Heidelberg University Hospital Department of Clinical Pharmacology and Pharmacoepidemiology Im Neuenheimer Feld 410 69120 Heidelberg Tel.: +49 (0) 6221 / 56- 38423 Fax: +49 (0) 6221 / 56- 8523 Email: felicitas.stoll@med.uni-heidelberg.de |
| **Biostatistician** | Prof. Dr. med. Dipl. Phys. Gerd Mikus  Heidelberg University Hospital Department of Clinical Pharmacology and Pharmacoepidemiology Im Neuenheimer Feld 410 69120 Heidelberg Tel.: +49 (0) 6221 / 56- 39197 Fax: +49 (0) 6221 / 56- 8523 Email: gerd.mikus@med.uni-heidelberg.de |

**SIGNATURE PAGE**

**The present trial protocol was subject to critical review and has been approved in the present version by the persons undersigned. The information contained is consistent with:**

- **the current risk-to-benefit assessment of the investigational medicinal products (IMPs)**
- **the moral, ethical, and scientific principles governing clinical research as set out in the applicable version of the Declaration of Helsinki and the principles of good clinical practice (GCP).**

**The investigator and all members of the trial group will be supplied with details of any significant or new finding including adverse events relating to treatment with the IMPs.**

**DECLARATION OF INVESTIGATOR**

**I have read the trial protocol and confirm that it contains all information to conduct the clinical trial. I pledge to conduct the clinical trial according to the protocol and according to the standard operating procedures of the Department of Clinical Pharmacology and Pharmacoepidemiology.**

**I will enrol the first participant only after all ethical and regulatory requirements are fulfilled. I will obtain written consent for trial participation from all participants after detailed oral and written information and according to the requirements of local law (AMG). According to GCP-V §7, Section 2 No 15, I declare that all trial participants will be informed on the type of encoding their personal data (pseudonymisation/anonymisation) and who receives or has access to such data. Participants who do not agree to this data encoding and transfer will not be enrolled into the trial. In this context I confirm (according to GCP-V §7, Section 3 No 15) that my investigational site complies with all local regulatory requirements for data protection.**

**Furthermore I declare (according to GCP-V §7, Section 3 No 4) that to the best of my knowledge no participants in a relationship of any dependence to the investigator or sponsor will be included.**

**I know the requirements for accurate notification of serious adverse events (SAE) and I pledge to document and notify such events as described in the protocol.**

**I declare that I am informed about the pharmacological-toxicological assessments and results regarding the benefits and risks of the clinical trial by reading the description in the clinical trial protocol and in the current version of the summary of product characteristics (SmPC). I ensure that all investigators/ relevant staff at my site will be informed of this results and possibly new risks that are forwarded by the sponsor later on (e.g. via new version of the SmPC ).**

**I confirm that every staff member will be adequately trained to guarantee compliance with the trial protocol incl. subsequent amendments.**

**I will retain all trial-related documents and source data as described. I will archive any trial-related documents according to the pertinent laws.**

**I will provide a Curriculum Vitae (CV) before trial start. I agree that the CV and Financial Disclosure (FD) may be submitted to the responsible ethical committee (EC).**

**As the clinical trial and the results have to be published in a clinical trial register and forwarded to the EC and competent authority. I agree that my name and clinic address will be part of this final trial (summary) report/ public register and are disclosed pursuant to §42b AMG.**

| **Principal Investigator’s Signature** | |
| --- | --- |
| **Investigator (”Prüfer”): Prof. Dr. med. Walter E. Haefeli** | |
| **Signature:** | **Date: 24.04.2020** |
|  | |

| **Deputy’s Signature** | |
| --- | --- |
| **Investigator (”Prüfer-Stellvertreter”): Dr. med. Antje Blank** | |
| **Signature:** | **Date: 24.04.2020** |
|  | |

| **Deputy’s Signature** | |
| --- | --- |
| **Investigator (”Prüfer-Stellvertreter”): Dr. med. Dipl. Vw. Felicitas Stoll** | |
| **Signature:** | **Date: 24.04.2020** |
|  | |

[1. Abbreviations 9](#_Toc37243402)

[2. Synopsis 11](#_Toc37243403)

[3. Introduction 19](#_Toc37243404)

[3.1. Background 19](#_Toc37243405)

[3.2. Trial rationale 19](#_Toc37243406)

[3.3. Risk-to-benefit ratio 20](#_Toc37243407)

[4. Trial objectives and endpoints 21](#_Toc37243408)

[4.1. Objectives 21](#_Toc37243409)

[4.1.1. Primary objective 21](#_Toc37243410)

[4.1.2. Secondary objectives 21](#_Toc37243411)

[4.1.3. Exploratory objectives 21](#_Toc37243412)

[4.2. Endpoints 21](#_Toc37243413)

[4.2.1. Primary endpoint 21](#_Toc37243414)

[4.2.2. Secondary endpoints 21](#_Toc37243415)

[4.2.3. Exploratory endpoints 22](#_Toc37243416)

[4.3. Outcome measures 22](#_Toc37243417)

[5. Trial design 22](#_Toc37243418)

[5.1. Clinical phase 22](#_Toc37243419)

[5.2. Trial design 22](#_Toc37243420)

[5.3. Overview 22](#_Toc37243421)

[5.4. Time schedule of the clinical trial 23](#_Toc37243422)

[6. Trial population 23](#_Toc37243423)

[6.1. Type and planned number of population 23](#_Toc37243424)

[6.2. Inclusion criteria 23](#_Toc37243425)

[6.3. Exclusion criteria 24](#_Toc37243426)

[6.4. Admission to the trial 24](#_Toc37243427)

[6.5. Withdrawal from treatment 24](#_Toc37243428)

[7. Trial procedures 24](#_Toc37243429)

[7.1. Visits and specific actions 25](#_Toc37243430)

[7.1.1. Screening visit 25](#_Toc37243431)

[7.1.2. Treatment period 25](#_Toc37243432)

[7.1.3. End-of-trial visit: 26](#_Toc37243433)

[7.2. Trial procedures 26](#_Toc37243434)

[7.2.1. Physical examination, vital signs, and electrocardiogram 26](#_Toc37243435)

[7.2.2. Blood and urine sampling, and total amount of blood withdrawn 27](#_Toc37243436)

[7.2.3. Metabolic genotypes 27](#_Toc37243437)

[Volunteers will participate in a genotyping study (K093). Evaluated genotypes of CYP enzymes will be documented and used in the present study as subjects’ characteristics. 27](#_Toc37243438)

[7.2.4. Priority and sequence of trial procedures 27](#_Toc37243439)

[7.3. Hospitalization / emergency visit 28](#_Toc37243440)

[7.4. Concomitant medication 28](#_Toc37243441)

[7.5. Dietary and other restrictions 28](#_Toc37243442)

[7.6. Assessment and recording of AEs 28](#_Toc37243443)

[8. Analytical procedures 29](#_Toc37243444)

[8.1. Quantification of drug concentrations in whole blood, plasma, and PBMC 29](#_Toc37243445)

[8.2. Labelling and storage of biological samples 29](#_Toc37243446)

[9. Trial medication – investigational medicinal products 30](#_Toc37243447)

[9.1. Hydroxychloroquine 30](#_Toc37243448)

[9.1.1. Pharmacological properties 30](#_Toc37243449)

[9.1.2. Packaging, labelling, and supply 31](#_Toc37243450)

[9.1.3. Administration of trial medication 31](#_Toc37243451)

[9.1.4. Adherence and drug accountability 31](#_Toc37243452)

[9.1.5. Safety and tolerability 31](#_Toc37243453)

[9.1.6. Contraindications 33](#_Toc37243454)

[9.2. Pantoprazole 33](#_Toc37243455)

[9.2.1. Pharmacological properties 34](#_Toc37243456)

[9.2.2. Packaging, labelling, and supply 34](#_Toc37243457)

[9.2.3. Administration of trial medication 34](#_Toc37243458)

[9.2.4. Adherence and drug accountability 34](#_Toc37243459)

[9.2.5. Safety and tolerability 35](#_Toc37243460)

[9.2.6. Contraindications 36](#_Toc37243461)

[9.3. Midazolam 36](#_Toc37243462)

[9.3.1. Pharmacological properties 36](#_Toc37243463)

[9.3.2. Packaging, labelling, and supply 37](#_Toc37243464)

[9.3.3. Administration of trial medication 37](#_Toc37243465)

[9.3.4. Adherence and drug accountability 37](#_Toc37243466)

[9.3.5. Safety and tolerability 37](#_Toc37243467)

[9.3.6. Contraindications 38](#_Toc37243468)

[9.4. Yohimbine 39](#_Toc37243469)

[9.4.1. Pharmacological properties 39](#_Toc37243470)

[9.4.2. Packaging, labelling, and supply 39](#_Toc37243471)

[9.4.3. Administration of trial medication 39](#_Toc37243472)

[9.4.4. Adherence and drug accountability 40](#_Toc37243473)

[9.4.5. Safety and tolerability 40](#_Toc37243474)

[9.4.6. Contraindications 40](#_Toc37243475)

[10. Assignment of participant codes 41](#_Toc37243476)

[10.1. Blinding and unblinding 41](#_Toc37243477)

[10.2. Randomization 41](#_Toc37243478)

[11. Monitoring 41](#_Toc37243479)

[12. End of trial 42](#_Toc37243480)

[12.1. Regular trial end 42](#_Toc37243481)

[12.2. Premature end of trial 42](#_Toc37243482)

[12.2.1. Early termination of the trial 42](#_Toc37243483)

[12.2.2. Stopping criteria for the trial 42](#_Toc37243484)

[12.3. Withdrawal of participants 42](#_Toc37243485)

[12.4. Dropouts 42](#_Toc37243486)

[13. Data analysis 43](#_Toc37243487)

[13.1. Sample size and power calculation 43](#_Toc37243488)

[13.2. Statistical analysis plan 43](#_Toc37243489)

[13.2.1. Analysis sets 43](#_Toc37243490)

[13.2.2. PK parameters 43](#_Toc37243491)

[13.2.3. Statistical analysis 43](#_Toc37243492)

[13.2.4. Procedure for handling missing, unused, and inconsistent data 44](#_Toc37243493)

[14. Ethic and legal aspects 44](#_Toc37243494)

[14.1. General 44](#_Toc37243495)

[14.2. Financial disclosure of investigator 44](#_Toc37243496)

[14.3. Submission to the Ethics Committee and Competent Authority (BfArM) 44](#_Toc37243497)

[14.4. Notification of authorities 44](#_Toc37243498)

[14.5. Responsibilities 45](#_Toc37243499)

[14.6. Insurance 45](#_Toc37243500)

[14.7. Confidentiality 45](#_Toc37243501)

[14.8. Archiving 46](#_Toc37243502)

[14.9. Protocol modifications and amendments 46](#_Toc37243503)

[14.10. Access to source data, quality control, and data handling 46](#_Toc37243504)

[14.11. Participant information and informed consent 47](#_Toc37243505)

[14.12. List of participants (participants’ log) 47](#_Toc37243506)

[14.13. Participant medical records / source data 47](#_Toc37243507)

[14.14. Data management 48](#_Toc37243508)

[14.15. Financing 48](#_Toc37243509)

[14.16. Publication of trial results 48](#_Toc37243510)

[15. References 49](#_Toc37243511)

[16. Appendix 50](#_Toc37243512)

[16.1. Definition of AEs 50](#_Toc37243513)

[16.2. Responsibilities of the PI 55](#_Toc37243514)

[16.3. Contraindicated drugs 56](#_Toc37243515)

# Abbreviations

| AE | Adverse event |
| --- | --- |
| AMG | (German) Arzneimittelgesetz |
| aPTT | Activated partial thromboplastin time |
| AUC | Area under the concentration-time curve |
| AUC_0-∝_ | Area under the concentration-time curve extrapolated to infinity |
| BfArM | Bundesinstitut für Arzneimittel und Medizinprodukte (Federal Institute for Drugs and Medical Devices) |
| CK | Creatine kinase |
| C_max_ | Maximum concentration |
| Cl | Clearance |
| Crea | Creatinine |
| CRF | Case report form |
| CTCAE | Common Terminology Criteria for Adverse Events |
| CYP | Cytochrome P450 isozyme |
| d | Day(s) |
| DSGVO | Datenschutzgrundverordnung (General Data Protection Regulation of the EU) |
| ECG | Electrocardiogram |
| eCRF | Electronic case report form |
| EOT | End-of-trial visit |
| FDA | (US) Food and Drug Administration |
| GCP | Good Clinical Practice |
| GCP-V | Good Clinical Practice - Act  Verordnung über die Anwendung der Guten Klinischen Praxis bei der Durchführung von klinischen Prüfungen mit Arzneimitteln zur Anwendung am Menschen |
| h | Hour(s) |
| HCQ | Hydroxychloroquine |
| HIV | Human immunodeficiency virus |
| HR | Heart rate |
| ICH | International Council for Harmonisation of Technical Requirements for Pharmaceuticals for Human Use |
| IMP | Investigational medicinal product |
| INN | International Nonproprietary Name |
| INR | International normalized ratio |
| KliPS | Klinisch-Pharmakologisches Studienzentrum (Clinical Research Unit) |
| min | Minute(s) |
| MS/MS | Tandem mass spectrometry |
| PI | Principal investigator |
| p.o. | Per os, orally |
| PK | Pharmacokinetic(s) |
| PPI | Proton-pump inhibitor |
| s | Second(s) |
| SAE | Serious adverse event |
| SCR | Screening visit |
| SmPC | Summary of product characteristics |
| SOP | Standard operating procedure |
| SUSAR | Suspected unexpected serious adverse reaction |
| T_max_ | Time to reach maximal concentration |
| t_1/2_ | Half-life |
| TSH | Thyroid stimulating hormone |
| ULN | Upper limit of normal |
| UPLC | Ultra-performance liquid chromatography |
| WNCBP | Women not of child-bearing potential |
| y | Year(s) |
| γ-GT | Gamma-glutamyltranspeptidase |

# Synopsis

| **Title** | **Impact of pantoprazole on absorption and disposition of hydroxychloroquine, a drug used in Corona Virus Disease-19 (Covid-19)** |
| --- | --- |
| **Short Title** | **PPI-HCQ** |
| **Clinical Trial Code** | K724 |
| **EudraCt No** | 2020-001470-30 |
| **Indication** | Drug-drug interaction (DDI) study |
| **Trial phase** | I |
| **Trial population** | A maximum of up to 36 volunteers may be included; 24 volunteers, 12 per arm, will be included; up to 12 volunteers may be replaced if enrolled volunteers drop out prematurely. . |
| **Objectives** | Primary objective   - Evaluation of the effect of the proton-pump inhibitor (PPI) pantoprazole on the absorption of HCQ in healthy volunteers.   Secondary objectives   - Comparison of HCQ concentrations in whole blood as compared to plasma and intracellular concentration as measured in target cells (peripheral blood mononuclear cells, PBMCs). - Evaluation of HCQ as a perpetrator drug in DDI at the level of cytochrome P450 (CYP) 3A and CYP2D6   Exploratory objectives  Evaluation of the effect of pantoprazole on the disposition of major HCQ metabolites |
| **Trial endpoints** | **Primary endpoint**   - AUC_0-72 h_ and C_max_ of a single oral dose of 400 mg HCQ with and without pantoprazole.   **Secondary endpoints**   - AUC_2-4 h_, AUC_0-6 h_, and C_max_ of microdosed midazolam and microdosed yohimbine, - Correlation of concentrations of HCQ in whole blood with concentrations in plasma and PBMCs.   **Exploratory endpoints**   - AUC_0-72 h_ and C_max_ of major metabolites of HCQ; this may include but is not limited to desethyl-HCQ with and without pantoprazole. |
| **Outcome measures:** | - Concentration-time curve of HCQ and derived PK parameters in whole blood, - Concentration-time curve of midazolam and yohimbine in plasma, - Concentrations of HCQ and desethyl-HCQ in plasma and PBMCs, - Concentration-time curve of HCQ metabolites and derived PK parameters. |
| **Trial design** | This is a single centre, open-label, parallel group, two-arm, one-sequence phase I DDI trial in healthy volunteers.  The result of the trial is expected to have impact on the HCQ treatment of COVID-19 patients, which is currently an urgent pandemic challenge. The parallel design is preferred to the cross-over design due to the very long elimination half-life of HCQ.  The trial consists of a screening visit (SCR), a treatment period, and an end-of-trial visit (EOT).  **Figure 1:** Trial design of a randomized two-arm, one sequence DDI trial in healthy volunteers evaluating the impact of pantoprazole therapy on HCQ PK. Concurrently, the relationship between HCQ PK and activities of CYP2D6 and CYP3A4 is investigated on the basis of exposure to the microdosed probe drugs yohimbine and midazolam (µMµY).  **Treatment period:**   \| Visit 1 (day 1): \| - Breakfast (3 h before drug administration) - Administration of 30 µg midazolam per os (p.o.), and - Administration of 50 µg yohimbine p.o. - Start 0-6 h pharmacokinetic assessments (PK) - After PK assessments start with pantoprazole (p.o.) for 9 d   Timing will be aligned with the expected timing of breakfast and dosing on V2. \| \| --- \| --- \| \| Visit 2 (day 6): \| - PPI therapy continued (morning dose 1 h before breakfast) - 400 mg HCQ p.o. with food - Start 0-24 h HCQ PK - 3 h after HCQ: administration of 30 µg midazolam and 50 µg yohimbine p.o. - Start 0-6 h midazolam and yohimbine PK \| \| Visit 3 – 5  (day 7-9): \| - PPI therapy continued until day 9 - PK sampling 24, 36, 48, and 72 h after HCQ administration \|   **EOT:** Assessment of the volunteers’ health at the end of the trial.  Telephone safety follow-up 90 d |
| **Investigational medicinal product** | 1. HCQ: 400 mg, single oral dose (V2) 2. Pantoprazole: 40 mg oral dosing, daily for 9 d (visits 1-5)   Pantoprazole will be taken after PK sampling on V1. Pantoprazole will be taken 1 h before a meal, usually breakfast. On V2/day 6 pantoprazole will be taken 1 h before breakfast and HCQ will be taken together with breakfast. On days 2-5, and 7-9 pantoprazole must be taken at home 1 h before breakfast.  Microdosing for CYP phenotyping will be performed at V1 and V2:   1. Midazolam 30 µg oral dosing 2. Yohimbine 50 µg (2 tablets 25 µg each) oral dosing   Microdoses will be given 3 h after breakfast/HCQ intake. Volunteers will not be allowed to eat for 2 more hours. Timing for administrationand dosing for V1 and V2 will be aligned. |

| **Pharmaco­kinetic sampling** | Midazolam and yohimbine (plasma): predose, 0.5 h, 1 h, 2 h, 2.5 h, 3 h, 4 h, and 6 h.  HCQ (lithium-heparin whole blood and plasma): predose, q20 min until 6 h, q 30 min until 8 h, q1h until 12 h, thereafter at 24 h, 36 h, 48 h, and 72 h.  PBMCs (sodium citrate cell preparation tube): 2h, 4 h, and 24 h (after HCQ). |
| --- | --- |
| **Inclusion criteria** | 1. Age 18-60 y inclusive at the time of consent, 2. Males and females of child-bearing potential who are willing to use a highly effective method of contraception during the treatment and for 3 months after last administration of the IMP or women not of child-bearing potential (WNCBP) or individuals who are convincingly sexually abstinent.   *Accepted methods of contraception are: combined hormonal contraception associated with inhibition of ovulation (oral, intravaginal, or transdermal), progestogen-only hormonal contraception associated with inhibition of ovulation (oral, injectable, or implantable), intrauterine device, intrauterine hormone-releasing system, bilateral tubal occlusion, vasectomized partner, or sexual abstinence.*  *WNCBP are defined as women who have been surgically sterilized (total hysterectomy or bilateral oophorectomy, bilateral tubal ligation, staples, or another type of sterilization) or who are postmenopausal for at least 2 y. Individuals who are convincingly sexually abstinent are also eligible.*   1. Understanding, ability, and willingness to fully comply with trial interventions and restrictions, 2. Willingness to participate in a genotyping study (K093), and 3. Ability to provide written, personally signed and dated informed consent to participate in the trial, in accordance with the International Conference on Harmonisation (ICH) Good Clinical Practice (GCP) Guideline E6, and applicable regulations, prior to any trial-related interventions. |
| **Exclusion criteria** | **At the time of screening:**   1. Clinically significant or relevant abnormalities in the medical history, physical examination, and laboratory evaluation as assessed by the investigator, 2. Any medical disorder that may require treatment or make the participant unlikely to fully complete the trial, or any condition that presents undue risk from the IMPs or trial interventions, 3. Clinically relevant ongoing or clinically relevant history of physical or psychiatric illness as judged by the investigator, especially a) Gastrointestinal, neurological, or haematological diseases b) history of psoriasis, porphyria, or epilepsy 4. Pregnancy or breast feeding, 5. Any acute or chronic illness or clinically relevant finding known or expected to modify absorption, distribution, metabolism, or excretion of HCQ, midazolam, yohimbine, or pantoprazole, 6. Any known history of severe allergic or anaphylactic reactions to drugs or food or any other clinically significant allergies (except mild forms of hay fever), 7. Any known allergies to the compound or further ingredients of HCQ, quinine, pantoprazole, midazolam, or yohimbine preparations, 8. Prolonged QTc time: women: QTcF > 460 ms, men: QTcF > 440 ms 9. Clinically relevant findings in any of the following investigations at SCR. Minor deviations of laboratory values from the normal range can be acceptable, if judged by the investigator to be of no clinical relevance for this trial: 10. Hemoglobin (Hb) < 12 g/dl (males) or < 11 g/dl (females), 11. Creatinine (Crea) clearance (Cl) < 60 ml/min (Cockcroft-Gault), 12. Bilirubin > upper limit of normal (ULN) x 1.2,  In case of suspected Gilbert’s disease: non-fasting total bilirubin ≤ ULN x 1.2 and fasting total bilirubin ≤ ULN x 1.5 are acceptable. 13. Alanine aminotransferase (ALT) > ULN x 1.1, 14. Aspartate aminotransferase (AST) > ULN x 1.2, 15. Creatine kinase (CK) not within normal limits (volunteers with CK elevations between ULN and ULN x 3 may be included if troponin T is negative, and 16. Thyroid stimulating hormone (TSH) not within normal limits, 17. A positive human immunodeficiency virus and hepatitis C antibody screen, 18. A positive result in the drug screening test (at SCR, see section 7.2.2) 19. Any intake of HCQ, chloroquine or travel to malaria risk regions within the last 3 months, 20. Use of any medication (prescription medication, non-prescription medication including multivitamin or herbal preparations) with active ingredients except hormonal contraception and thyroid hormones, or any intake of substances known to induce or inhibit HCQ-metabolizing enzymes or drug transporters within a period of less than 5 times the respective elimination half-life (t_1/2_) with regard to the expected date of the first dose of IMP, 21. Consumption of citrus fruits or products of these fruits within 7 d prior to the expected date of first dose of IMP and expected nonadherence to refrain from such products until V5, 22. Expected nonadherence to refrain from alcohol 24 h prior to V1 until V5 of this trial, or excessive alcohol consumption. 23. Intake of quinine, or consumption of quinine-containing drinks (bitter lemon, tonic water, bitter orange) 24. Use of an IMP within 30 d prior to the expected date of receiving the first dose of IMP or active enrolment in another drug or vaccine clinical trial. 25. Contraindications to HCQ use: 26. Hypersensitivity to the active substance, 4-aminoquinoline, or to any of the excipients 27. History of retinopathy or maculopathy 28. Glucose-6-phosphat-dehydrogenase deficiency (fauvism, haemolytic anemia) 29. Diseases of the haematopoietic system 30. Myasthenia gravis 31. During pregnancy (except for treatment of malaria) 32. While breastfeeding   **At V1, prior to dosing:**   1. Use of any medication (prescription medication, non-prescription medication including multivitamin or herbal preparations) with active ingredients except hormonal contraception and thyroid hormones or any intake of substances known to induce or inhibit HCQ-metabolizing enzymes or drug transporters within a period of less than 5 times the respective elimination half-life (t1/2) with regard to the expected date of the first dose of IMP, and 2. Use of an IMP within 30 d prior to receiving the first dose of IMP or active enrolment in another drug or vaccine clinical trial. |
| **Statistics** | Planned sample size: of 24 healthy volunteers, 12 in each arm.  The sample size calculation is based on previous data after single dose of HCQ [1]. An online calculator was used for the sample size calculation (http://hedwig.mgh.harvard.edu/sample_size/js/js_parallel_quant.html). With the AUC_0-72 h_ data available, a sample size of 14 (7 in each group) is needed to detect a 30 % difference in AUC_0-72 h_ with a power of 0.8 and an α-error of 5 %. Therefore, a sample size of 12 per group is regarded as sufficient to detect a PPI-induced difference on HCQ AUC_0-72 h_ exceeding the bioequivalence range (80-125 %).  Up to 12 volunteers may be replaced if enrolled volunteers drop out prior to exposure with HCQ or prior to completing the 72 h PK sampling period. |
| **Timelines** | Approved protocol 2^nd^ quarter 2020  First participant enrolled 2^nd^ quarter 2020  Last participant last visit Q III 2020  Data base lock Q IV 2020  End of trial report 2^nd^ quarter 2021 |

**Table 1:** Trial procedures

| Visit | **Screening** | 1 | 2 | 3 | 4 | 5 | EOT | Safety call |
| --- | --- | --- | --- | --- | --- | --- | --- | --- |
| **Trial day** (h/day in relation to exposure) | **- 28 to -1** | **1** | **6** (0-12 h) | **7** (24 ± 1, 36 ± 1 h) | **8** (48 ± 2 h) | **9** (72 ± 2 h) | **+7 to + 28** (day 16 - 37) | (day 90-120) |
| Participant information and informed consent | X |  |  |  |  |  |  |  |
| Inclusion / exclusion criteria | x |  |  |  |  |  |  |  |
| Relevant medical history / current medical condition | x |  |  |  |  |  |  |  |
| Co-medication | x | x |  |  |  |  |  |  |
| Physical examination | x |  |  |  |  |  | x |  |
| Haematology, blood chemistry^1^ | x | x |  |  |  |  | x |  |
| Urinanalysis | x |  |  |  |  |  | x |  |
| Pregnancy test (female volunteers) | x |  |  |  |  |  | x |  |
| HCV and HIV serology | x |  |  |  |  |  |  |  |
| Drug screen | x |  |  |  |  |  |  |  |
| 12-lead electrocardiogram | x |  | x (4 h) |  |  |  | x |  |
| **Vital signs and body measurements:** |  |  |  |  |  |  |  |  |
| Body height | x |  |  |  |  |  |  |  |
| Body weight | x |  |  |  |  |  | x |  |
| Body temperature | x |  |  |  |  |  | x |  |
| Blood pressure / heart rate | x | x | x^2^ |  |  |  | x |  |
| Adverse Events |  | x | x | x | x | x | x | x |
| **Drugs administered p.o.:** |  |  |  |  |  |  |  |  |
| 400 mg HCQ |  |  | x |  |  |  |  |  |
| 40 mg pantoprazole |  | x | x | x | x | x |  |  |
| 30 µg midazolam |  | x | x |  |  |  |  |  |
| 50 µg yohimbine (2 x 25 µg) |  | x | x |  |  |  |  |  |
| **Blood collections:** |  |  |  |  |  |  |  |  |
| PK blood collection (HCQ)^3^ |  |  | x | x | x | x |  |  |
| PK plasma collection (midazolam, yohimbine)^4^ |  | x | x |  |  |  |  |  |
| PBMC^5^ |  |  | x | x |  |  |  |  |

EOT end-of-trial visit, HCV hepatitis C virus, HIV human immunodeficiency virus, PBMC peripheral blood mononuclear cells, PK pharmacokinetics, p.o. per os/oral intake.

1: Red blood-cell count, haematocrit, haemoglobin, white blood-cell count with differentials (neutrophils, eosinophils, basophils, monocytes and lymphocytes), platelets. Electrolytes (sodium, potassium, calcium, chloride, phosphate), aspartate aminotransferase, alanine aminotransferase, alkaline phosphatase, gamma-glutamyltranspeptidase, creatine kinase, troponin T, total bilirubin, creatinine, creatinine clearance (Cockcroft-Gault), total protein, albumin, lipase, and blood glucose.
International normalized ratio, activated partial thromboplastin time.

2: Predose, q3 h on V1 and V2.

3: V2: predose, until 6 h post-dose: every 20 min; from 6-8 h post-dose: every 30 min; from 8,12 h post-dose: every hour. V3: 24 h and 36 h post-dose. V4: 48 h post-dose. V5: 72 h post-dose.

4: V1, V2: predose, 0.5 h, 1 h, 2 h, 2.5 h, 3 h, 4 h, and 6 h after administration.

5: 2 h, 4 h, and 24 h post-dose.

# Introduction

## Background

**Description of scientific background**

The search for effective therapy of Corona Virus Disease-19 (COVID-19) has put HCQ into the focus of interest. In the absence of proven effective drugs, explorative treatment schedules for COVID-19 patients now foresee the use of oral HCQ although the risk-benefit ratio remains currently unclear. Particularly in seriously ill patients and elderly patients, PPI therapy for prophylaxis or treatment of gastric or duodenal ulcers or gastroesophageal reflux disease is common. Bioavailability of oral HCQ is highly variable and reported values range between 30-70 %. With acid dissociation constant (pKa) values of 9.67 and 8.27, HCQ crosses membranes readily at neutral pH but accumulates in acidic compartments where it is protonated and thus charged and better soluble. Therefore, changes in gastric pH could be associated with alterations of HCQ bioavailability and exposure because of impaired solubility and this could translate into variability of treatment success.


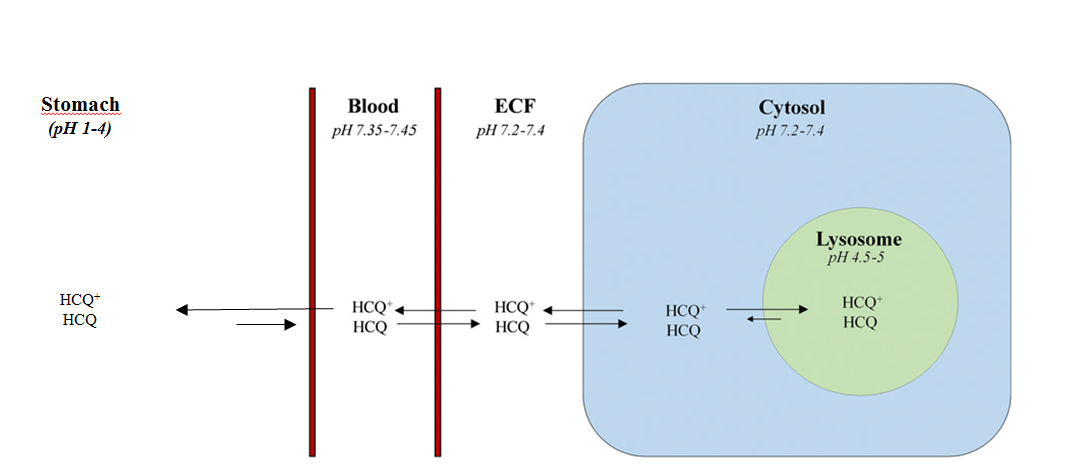


**Figure 2:** HCQ PK is pH-dependant (modified from J Pharmacol Exp Ther 2018;365:447 [1]; *ECF extracellular fluid, HCQ hydroxychloroquine*).

## Trial rationale

**Description of scientific rationale**

We hypothesize that absence and presence of PPI could modulate the outcome of HCQ therapy and unintended low HCQ (or active metabolite) blood concentrations could be related to an unfavourable course of disease, as observed in patients with systemic lupus erythematosus [2-4]. In particular, we expect that in the absence of acid-modifying therapies oral HCQ will be trapped in the acidic environment of the stomach whereas PPI therapy will increase pH and thus render the compound more lipophilic and hence foster absorption. In addition, HCQ could act as a perpetrator and victim at CYP3A, CYP2D6, and possibly also of the CYP2C family.

This trial will investigate the potential effect of pantoprazole on HCQ absorption in vivo. HCQ blood concentrations will be measured in participants under pantoprazole treatment, and will be compared to participants who do not receive PPI or other antacid.

Furthermore, we aim to characterize the relationship between relevant CYP activities metabolic profile of HCQ by administering a microdose of midozolam, and yohimbine.

## Risk-to-benefit ratio

**Description of risk - benefit balance**

Hydroxychloroquine (HCQ) is an approved drug for adults and children used in malaria prophylaxis and therapy, as well in the treatment of rheumatic diseases (i.e. lupus erythematodes, and rheumatoid arthritis). In malaria prophylaxis, the recommended dose for adults is 400 mg once a week, starting two weeks before travelling into areas with endemic risk. In the treatment of rheumatic diseases, a starting dose of up to 600 mg per day can be administered. [5]

In our trial, participants will receive a single dose of 400 mg. The risk of adverse events (AEs) (summarized in section 9.1.5) is expected to be low. Safety parameters will be closely monitored. Considering the long terminal elimination half-life of HCQ, an additional telephone safety follow-up after 90 d will be performed.

Pantoprazole is a PPI licensed since more than 2 decades. It reduces secretion of gastric acid and is widely used for prophylaxis and treatment of reflux esophagitis or gastric ulcer. In those indications, the recommended dose is 40 mg (80 mg in individual cases) per day for 4 to 8 weeks. In Zollinger-Ellison syndrome, the duration of therapy is unlimited and short-term dose increases of up to 160 mg daily may be required. [6]

In our trial, the participants in one arm will take pantoprazole in a standard dose of 40 mg for 9 d. The risk of AEs (summarized in section 9.1.5) is expected to be low. Safety parameters of interest, such as transaminases, will be monitored.

The CYP3A activity will be phenotyped by using a pharmacologically inactive dose of 30 µg midazolam as a diagnostic substance. This dose is over 100 times lower than the regular doses used in a therapeutic setting and no benzodiazepine effects are to be expected [7, 8]. Therefore, the risks of this procedure are negligible.

The CYP2D6 activity will be phenotyped by using a pharmacologically inactive dose of 50 µg yohimbine as a diagnostic substance. No pharmacological effects are to be expected [9]. Therefore, the risks of this procedure are negligible.

Blood samples will be taken from a peripheral venous catheter. Insertion and drawing are generally well tolerated by healthy subjects. Occasionally, venipuncture or the placement of an indwelling catheter may result in thrombosis or thrombophlebitis and/or peripheral nerve damage (numbness). A total amount of approximately 400 ml will be drawn over the course of at least 17 d. This blood loss is generally well tolerated.

In summary it is unlikely that severe AEs will result from the single dose administration of HCQ or the administration of pantoprazole, as well as from carrying out the procedures of the trial. This trial will be conducted in the ISO9001-certified Clinical Research Unit of the Heidelberg University Medical Centre. Emergency equipment and medication are at hand and an intensive care unit is within reach. The medical staff is trained for cardio-respiratory emergency situations. Before including individuals in this trial, we will perform a complete health check, including vital signs, physical examination, laboratory assessment, and electrocardiogram (ECG). Inclusion and exclusion criteria will be checked. Any clinically relevant pathological finding will lead to exclusion. Vital parameters of all volunteers are monitored during their in-house visit. All relevant safety parameters including safety laboratory parameters and ECGs will be closely monitored during the trial.

HCQ is currently administered to severely ill COVID-19 patients. HCQ dose schemes vary between institutions, but an initial 400 mg dose appears advisable in order to achieve high concentrations rapidly [10]. Significant reductions of blood concentrations could have an impact on treatment response, but also on the systematic evaluation of treatment results. Advancing the treatment concepts of COVID-19 would bring a relevant benefit to society. Therefore, the exposure of healthy volunteers to HCQ and pantoprazole is justified, although no direct benefit for these participants is expected.

# Trial objectives and endpoints

## Objectives

- - 1. Primary objective
- Evaluation of the effect of the PPI pantoprazole on the absorption of HCQ in healthy volunteers.
  - 1. Secondary objectives
- Comparison of HCQ concentrations in whole blood as compared to plasma and intracellular concentration as measured in target cells (peripheral blood mononuclear cells, PBMCs).
- Evaluation of HCQ as a perpetrator drug in DDI at the level of CYP3A and CYP2D6
  - 1. Exploratory objectives
- Evaluation of the effect of pantoprazole on the disposition of major HCQ metabolites

## Endpoints

- - 1. Primary endpoint
- AUC_0-72 h_ and C_max_ of a single oral dose of 400 mg HCQ with and without pantoprazole.
  - 1. Secondary endpoints
- AUC_2-4 h_, AUC_0-6 h_ and C_max_ of microdosed midazolam and microdosed yohimbine,
- Correlation of concentrations of HCQ in whole blood with concentrations in plasma and PBMCs.
  - 1. Exploratory endpoints
- AUC_0-72 h_ and C_max_ of major metabolites of HCQ, this may include but is not limited to desethyl-HCQ with and without pantoprazole.

## Outcome measures

- Concentration-time curve of HCQ and derived PK parameters in whole blood,
- Concentration-time curve of midazolam and yohimbine in plasma,
- Concentrations of HCQ and desethyl-HCQ in plasma and PBMCs,
- Concentration-time curve of HCQ metabolites and derived PK parameters.

# Trial design

## Clinical phase

Phase I (DDI trial).

## Trial design

This is a single centre, open-label, parallel group, two-arm, one-sequence phase I DDI trial in healthy volunteers.

The result of the trial is expected to have impact on the HCQ treatment of COVID-19 patients, which is currently an urgent pandemic challenge. The parallel design is preferred to the cross-over design due to the very long elimination half-life of HCQ.

The trial consists of a SCR visit, a treatment period, and an EOT visit.

## Overview

**Figure 3:** Trial design of a randomized two-arm, one sequence DDI trial in healthy volunteers evaluating the impact of pantoprazole therapy on HCQ PK. Concurrently, the relationship between HCQ PK and activities of CYP2D6 and CYP3A4 is investigated on the basis of exposure to the microdosed probe drugs yohimbine and midazolam (µMµY).

The trial duration for each participant can vary between 17 and 65 d, the screening period may last up to 28 d and will be followed by a minimum 9 d treatment period. The EOT is planned 7 to 28 d after V5.

## Time schedule of the clinical trial

See synopsis.

EOT is defined as the date of database lock.

# Trial population

## Type and planned number of population

A minimum of 24 healthy volunteers (12 per arm) will be included. In total, a maximum of up to 36 volunteers may be included.

Gender is not expected to have an impact for the evaluation of the DDI. Therefore female and male participants will be included in an undefined proportion.

## Inclusion criteria

See synopsis.

## Exclusion criteria

See synopsis

## Admission to the trial

If all inclusion criteria are met and no exclusion criterion is present, a volunteer will be enrolled in the trial after clinical, medical, and laboratory examinations have been evaluated in a positive manner at SCR. Starting on admission on the first visit, participants have to report any changes in their health throughout the whole trial until the EOT visit. A physician who is a member of the trial team will have to certify that according to his/her knowledge the individual is healthy and fit for the trial by signing the statement of eligibility in the case report form (CRF) of the individual.

## Withdrawal from treatment

Any participant can withdraw from treatment at any time without personal disadvantages and without having to give a reason. Participants who discontinue exposure to the trial treatment on their own will be defined as premature withdrawals. Premature withdrawals will be replaced. The time of withdrawal from trial and/or treatment discontinuation must be documented in the source data and on the CRF.

The investigator can also discontinue the treatment after considering the risk-to-benefit ratio, e.g. if he/she considers that further treatment of the participant according to the trial protocol is no longer justifiable. The date of and the primary reason for the withdrawal from treatment, as well as the observations available at the time of withdrawal will be documented in the CRF.

Reasons leading to the withdrawal of a participant can include but are not limited to the following: intolerable AE, lack of participant’s cooperation, new occurrence of a disease precluding continuation, emergence of major exclusion criteria (coming to light after inclusion) that potentially endanger the safety of the participant.

If consent is not withdrawn, every effort must be made to obtain safety parameters until the EOT visit.

# Trial procedures

The trial will be conducted in the Department’s clinical trial unit (Klinisch-Pharmakologisches Studienzentrum, Clinical Research Unit; KliPS), which is certified according to DIN EN ISO9001. All interested trial participants will be provided with the written trial information at least one day prior to the oral information and will be given enough time to discuss the trial with a trial physician before or on the day of the SCR. After signing the informed consent form, trial procedures will start. Inclusion and exclusion criteria will be checked, and each participant will undergo a standardised series of interventions according to the trial schedule (Table 1).

## Visits and specific actions

- - 1. Screening visit

Each participant will undergo a SCR within 4 weeks before the first trial day. All inclusion and exclusion criteria will be checked. The relevant medical history and medical condition will be assessed. Additionally, a complete physical examination (including body height, weight, body temperature (T), and blood pressure (BP)/heart rate (HR)), urine, and blood analysis (haematology, blood chemistry, coagulation, HIV, and hepatitis B/C), and an ECG evaluation will be done.

Abnormal parameters at SCR may be re-tested if thought to be a measurement error or to be the result of a transient and reversible condition. The last obtained value will define eligibility. At the end of the SCR, all inclusion and exclusion criteria must be evaluated and a decision will be taken if the volunteer can be included into the trial.

| **Visits** | **Action** |
| --- | --- |
| **SCR** | - Medical history, - Confirmation of inclusion and exclusion criteria, - Physical examination, vital signs, height, and body weight, - ECG evaluation, - Blood sampling (haematology, blood chemistry, and HCV and HIV serology). - Urinalysis, pregnancy test, and screening for illicit drugs (see 7.2.2). |

If a participant cannot be enrolled into the trial for administrative reasons, rescreening may occur by repeating the screening visit.

- - 1. Treatment period

During the treatment period, the participants are expected to be present in the unit from approximately 30 min to 1 h prior to any activity to approximately 15 min after the scheduled trial procedure of a visit. This signifies:

- On V1, participants will be confined to the study unit from approximately 10:00 a.m. for approximately 8 h.
- On V2, participants will be confined to the study unit from approximately 7:00 a.m. for approximately 15 h.
- On V3, participants will be visiting the study unit two times for approximately 1h for 24-h and 36-h PK blood sampling
- On V4 and V5, participants will visit the study unit once for 48-h and 72-h blood sampling, respectively.

| Visit 1 (day 1): | - Breakfast (3 h before drug administration) - Administration of 30 µg midazolam p.o. - Administration of 50 µg yohimbine p.o. - Start 0-6 h PK assessments - After PK assessments start with pantoprazol (p.o.) for 9 d   Timing will be chosen related to expected timing of breakfast and dosing on V 2. |
| --- | --- |
| Visit 2 (day 6): | - PPI therapy continued (morning dose 1 h before breakfast) - 400 mg HCQ p.o. with food - Start 0-24 h HCQ PK - 3 h after HCQ: administration of 30 µg midazolam and 50 µg yohimbine p.o. - Start 0-6 h midazolam and yohimbine PK |
| Visit 3 – 5  (day 7-9): | - PPI therapy continued until day 9 - 24 h ± 1, 36 h ± 1, 48 h ± 2 and 72 h ± 2 PK |

Unscheduled safety visits and tests may be performed as a consequence of AEs at the discretion of the investigator, if clinically indicated.

- - 1. End-of-trial visit:

The EOT must occur more than 7 d up to 28 d after HCQ administration and includes the following:

| **EOT** | - Current medical condition, - Assessment of AEs, - Physical examination including BP, HR, T, and body weight, - ECG evaluation, - Blood sampling (haematology, blood chemistry, safety lab, - Urinalysis, and - Confirmation of individual trial end or assessment of need to follow up any open AEs. |
| --- | --- |

Additionally, there will be a telephone safety on day 90 - 120 after HCQ administration.

## Trial procedures

Procedures will be carried out according to the Department’s standard operating procedures (SOPs).

- - 1. Physical examination, vital signs, and electrocardiogram

The physical examination consists of a review of organ systems including lungs, heart, abdomen, liver, kidneys, peripheral pulses, eyes, nose, throat, skin, and neurological status.

Vitals sign assessment consists of measurement of BP, HR, and T. Height and weight will be measured where indicated.

ECGs will be carried out as 12-lead ECGs.

- - 1. Blood and urine sampling, and total amount of blood withdrawn

Blood sampling will be done as shown in Table 1. Blood sampling at 24 h and 48 may occur with a deviation of ± 1 h and at 72 h it may occur with a deviation of ± 2 h. The total amount of blood will be approximately 400 ml withdrawn over the trial period (including SCR and EOT examinations). If rescreening occurs, an additional 30 ml of blood will be taken.

**Safety laboratory assessments**

These assessments will be carried out at the central lab of Heidelberg Medical Centre according to the routine hospital procedures.

- Haematology: red blood cell count, haematocrit, haemoglobin, white blood cell count with differentials (neutrophils, eosinophils, basophils, monocytes, and lymphocytes), platelets,
- Electrolytes/biochemistry: sodium, potassium, calcium, chloride, phosphate, AST, ALT, alkaline phosphatase (AP), gamma-glutamyltransfeptidase (γ-GT), CK, troponin T, total bilirubin, Crea, Crea Cl (Cockcroft-Gault),total protein, albumin, lipase, and plasma glucose,
- Coagulation: international normalized ratio (INR), activated partial thromboplastin time (aPTT) (at SCR only),
- Urinalysis: dip-stick test with optional microscopic examination (as per routine of the central laboratory), pregnancy test, and drug screen test (dip stick for amphetamines, barbiturates, benzodiazepines, cocaine, methamphetamines, ecstasy (MDMA), opiates, methadone, TCA (tricyclic antidepressants)).

In agreement with the hospital routine, samples measured at the central laboratory will be destroyed 48 h after analysis.

**Drug concentrations:**

There will be whole blood samples, plasma samples and PBMC samples for quantification of drug concentrations. All samples will be frozen at least at -20 °C or lower and will be analysed as outlined in section 6.

- - 1. Metabolic genotypes

Volunteers will participate in a genotyping study (K093). Evaluated genotypes of CYP enzymes will be documented and used in the present study as subjects’ characteristics.

- - 1. Priority and sequence of trial procedures

In case of coinciding activities (activities that are scheduled at the same time of the clinical trial), the activities will be performed in the following order:

- Blood sampling,
- Clinical observations/examinations,

The administration of pantoprazole will be 1 h prior to the oral intake of HCQ. HCQ is taken together with a meal (standardized Breakfast). The diagnostic substances midazolam and yohimbine will be dosed 3 h after breakfast/HCQ intake at the same time, which is defined as administration within 60 seconds (s). Deviations up to + 60 s are acceptable. If intake of the drugs deviates further, the blood drawing time points must relate to the intake of the respective drug.

## Hospitalization / emergency visit

No hospitalizations are planned during this trial. Participants who are admitted to a hospital or have to attend an emergency unit unexpectedly will be requested to inform the trial centre as soon as possible. However, an overnight stay in the study unit may be allowed following the day of V2/ day 6 for organizational reasons.

## Concomitant medication

No other medication (prescription medication, over-the-counter medicine including multivitamin, or herbal) should be taken during the trial period to exclude potential DDI, except hormonal contraception and levothyroxine or, if medically indicated, for intercurrent disease.

Contraindicated drugs are mentioned in the SmPC (see Appendix 16.3).

## Dietary and other restrictions

Participants will have to refrain from intake of citrus fruits or products of these fruits during the course of the trial.

Participants will have to arrive in the fasted state (at least 6 h fasted) on all trial visits except for screening. During V2, a standard meal will be served to the participants, which is to be eaten at the time of the intake of HCQ.

Because we do not expect any pharmacodynamic action from the used diagnostic administration of a midazolam microdose, there will be no restrictions on activities requiring complete mental alertness, such as working with hazardous machineries or driving a motor vehicle.

## Assessment and recording of AEs

Any AE must be documented and suspected unexpected serious adverse reactions (SUSAR) will be reported to the responsible authorities (for definition see Appendix 16.1) such as the EC, the Federal Institute for Drugs and Medical Devices (BfArM), and the Eudra Vigilance Database. In all cases, the EU-report form must show the identity of the investigator and be dated and signed. The description of the AE will include the time of onset, duration, seriousness, intensity, outcome, relationship to the study drug and any treatment required. The investigator will assess the intensity and causality of any AE (for classification and assessment see Appendix 1).

In this trial, the overnight stay at V2 does not qualify as SAE.

The observation period begins with the first administration of the IMP (any AEs prior to the first administration of the IMP are documented as medical history) and ends with the EOT visit. The investigator follows the outcome of AEs or abnormal laboratory findings that are related to study activities until recovery or stabilisation of the participant’s state. Further therapeutic consequences will be documented, for example any medical treatment or drop-out of the study.

In the case of a SAE, the Safety Officer of the Department will be informed within 24 h. Notification on a SAE form can be handed over in person, sent by mail or fax, and will be documented.

Fax number: +49 (0) 6221 / 56-8523

Email: [SAE.KLIPS@med.uni-heidelberg.de](mailto:SAE.KLIPS@med.uni-heidelberg.de)

**Period of observation and documentation**

AEs will be ascertained by the investigators using non-leading questions, noted as spontaneously reported by the patients to the medical staff, or observed during any measurements on all study days. The observation period begins with the first administration of an IMP and ends at the end of study EOT visit. Any event prior to first administration of the IMP is documented as medical history. AEs will be documented in the patient´s file and in the CRF. All participants who present with AEs, whether considered associated with the use of the trial medication or not, will be monitored by the responsible investigator to determine their outcome; this applies also to withdrawals.

# Analytical procedures

## Quantification of drug concentrations in whole blood, plasma, and PBMC

All samples will be processed within 30 min after sampling and whole blood, plasma and PBMCs will be stored at least at ≤ -20 °C or lower. HCQ concentrations will be quantified in whole blood, plasma, and PBMCs based on ultra-performance liquid chromatography (UPLC) - tandem mass spectrometry (MS/MS). The method is currently under development. Midazolam and yohimbine will be quantified by UPLC-MS/MS [11, 12]. These methods are validated according to FDA standards [13, 14].

## Labelling and storage of biological samples

The labels of collected blood samples will only contain pseudonymised data of the participant. Plasma samples will be labelled with the trial code, trial information, and the specific participant code and kept until the analysis has been completed, up to a maximum of 10 y. They will be destroyed at the latest 15 y after the EOT. If the participant withdraws consent at any time point in the trial, he/she may contact the respective medical investigator and request that samples will be destroyed.

# Trial medication – investigational medicinal products

**Hydroxychloroquine**

Administration of 400 mg p.o. (single dose of two 200-mg tablets) on V2/day 6.

**Pantoprazole**

Administration of 40 mg p.o. q.d. on days 1-9.

**Midazolam**

Administration of 30 µg p.o. (single dose) on V1/day 1 and V2/day 6.

**Yohimbine**

Administration of 50 µg p.o. (single dose) on V1/day 1 and V2/day 6.

## Hydroxychloroquine

Quensyl®, 200 mg tablets, marketed drug available from Sanofi-Aventis Deutschland GmbH, 65926 Frankfurt am Main.

Table 2: HCQ drug information

| Proprietary name: | Quensyl® |
| --- | --- |
| International Nonproprietary Name (INN): | Hydroxychloroquine |
| ATC code, if officially registered: | P01BA02 |
| Manufacturer: | Sanofi-Aventis Deutschland GmbH, 65926 Frankfurt am Main |
| Pharmaceutical formulation: | Tablet |
| Dosage: | 2 x 200 mg = 400 mg (single dose) on V2 |
| Mode of administration: | Oral, together with a meal |
| Storage instructions: | No specific storage instructions |

- - 1. Pharmacological properties

HCQ belongs to the group of 4-aminoquinolines. By binding to porphyrine, 4-aminoquinolines exert their effect in the prophylaxis and treatment of malaria. The immunemodulatory effect of HCQ, important for its use in rheumatic diseases and presumably Covid-19, is probably mediated by inhibition of T-cell proliferation, the complement system, or antigen-antibody reactions.

HCQ is nearly completely absorbed reaching C_max_ after 4 h. Bioavailability is about 75 % [15]. In blood, HCQ is mainly bound to cells; in plasma, plasma protein binding is approximately 50 %. HCQ accumulates in blood cells and organs: The concentration found in pigmented cells, for instance, is up to 1000 times higher than in plasma. Half-life is 30 to 60 days, assuming a multi-compartmental model.

The main active metabolite of HCQ is desethyl-HCQ. It is mainly excreted via faeces with a smaller portion excreted into urine, which consists of 60 % of HCQ. [5]

- - 1. Packaging, labelling, and supply

The trial medication is a marketed product. There will be no trial-specific labelling for HCQ because this drug is approved for human use in Germany and will be used in unchanged marketed form.

The investigator will confirm correct receipt of the trial medication in writing and ensure that the medication is safely and correctly stored. The investigator will document the use of the trial medication. The site monitor will periodically check the supplies of trial medication held by the investigator to ensure the correct accountability of all trial medication used. At the EOT, all unused trial medication will be completely destroyed or may be transferred to third parties according to legal use, both options will be documented. It will be assured that a final report of the drug accountability is prepared and maintained by the investigator.

- - 1. Administration of trial medication

Participants will receive 400 mg of HCQ as a single oral dose. The administration of the trial drug will take place at the trial site, together with a meal. No dose adjustments are planned.

- - 1. Adherence and drug accountability

HCQ will be administered by the trial team; therefore, taking and timing adherence of 100 % is expected. A drug accountability log will document the use of drug.

- - 1. Safety and tolerability

After oral administration of HCQ, the AEs listed below are known. Notably, most AEs are dose-dependent and occur at plasma concentrations of > 250 µg/l [5].

Table 3: HCQ adverse drug reactions [5].

| Blood und lymphatic system | Bone marrow depression, anaemia, thrombocytopenia, leukopenia, agranulocytosis, pancytopenia, aplastic anaemia (all******).  Precaution due to findings from known chloroquine effects: Methaemoglobinaemia*****, eosinophilia (with lung infiltration) *****. |
| --- | --- |
| Immune system | Urticaria, angiooedema, bronchospasm (all******). |
| Endocrine disorders | Reduced appetite*, hypoglycaemia******.  Exacerbation of porphyria. |
| Psychiatric disorders | Labile affect**, sleep disturbances or sleepiness***, confusion***, unrestlessness***, paraesthesia***; exacerbation of psychosis******, suicide******. |
| Nervous system disorders | Headache**, dizziness***, seizures******, dystonia******, dyskinesia******, akathisia******, tremor******. |
| Eye disorders | Blurred vision (disturbed accommodation, dose-dependent and reversible)**, retinopathy (seems reversible in early stage, irreversible especially in long-term treatment; dose-dependent -> rare if daily dose ≤ 6.5 mg/kg body weight)***/*****, corneal changes (oedema, opacity; reversible)***, maculopathy/ macula degeneration******. |
| Ear and vestibular organ | Vertigo***, tinnitus***, deafness****** |
| Heart and vessels | Cardiomyopathy, including fatal heart failure******, depression of T wave******, conduction disorders (partially reversible after withdrawal of Quensyl®)******.  Precaution due to findings from known chloroquine effects: drop of blood pressure. |
| Gastrointestinal disorders | Abdominal pain*, nausea*, flatulence**, diarrhoea with weight loss**, vomiting** (usually reversible after dose reduction / withdrawal of Quensyl®). |
| Hepatobiliary disorders | Liver damage (change of transaminases and bilirubin)***, liver failure******. |
| Skin and subcutaneous tissue disorders | Pruritus**, rash**, disturbance of skin and hair pigmentation***, hair loss***.  Bullous rashes (incl. erythema multiforme, toxic epidermal necrolysis, Stevens-Johnson syndrome, exfoliative dermatitis), DRESS, photosensibilisation, acute generalized exanthematous pustulosis (all******).  Exacerbation of porphyria cutanea tarda (porphyrinuria) or of psoriasis****. |
| Musculoskeletal and connective tissue disorders | Sensomotoric disturbances***, myopathies/neuromyopathies (can be reversible)******. |
| Renal and urinary disorders | Precaution due to findings from known chloroquine effects: under long-term therapy phospholipidosis (reversible)*****. |

Frequency: *very common **common ≥ 1/100 to < 1/10, ***uncommon ≥ 1/1000 to < 1/100; **** rare ≥ 1/10.000 to < 1/1.000; *****very rare < 1/10.000; ******not known

- - 1. Contraindications

Quensyl® must not be taken in case of

- Hypersensitivity to the active substance, 4-aminoquinoline or to any of the excipients
- Retinopathy or maculopathy
- Glucose-6-phosphat-dehydrogenase deficiency (fauvism, haemolytic anaemia)
- Diseases of the haematopoietic system
- Myasthenia gravis
- During pregnancy (except for treatment of malaria)
- While breastfeeding
- Children under the age of 6 y (< 35 kg)
  - 1. Pregnancy precaution

HCQ is contraindicated during pregnancy unless given for the prophylaxis or treatment of malaria, which is an adverse disease itself for pregnant women. Epidemiological studies did not show a significantly increased risk for congenital malformation or negative course of pregnancy under HCQ. However, the structurally similar chloroquine showed an increased risk for congenital malformation, reproductive toxicity and genotoxicity. The manufacturer requests contraception during therapy and at least 3 months after end of therapy. As HCQ is the drug with the highest prenatal toxicity risk, the requirements for HCQ are the leading requirements for pregnancy prevention in this trial. Therefore volunteers must practice adequate contraception with a highly effective measure (women) and an effective measure (men) for the duration of the study and at least 3 months thereafter.

## Pantoprazole

Pantoprazol HEXAL, 40 mg tablets, marketed drug available from Hexal AG, Industriestraße 25, 83607 Holzkirchen.

Table 4: pantoprazole drug information

| Marketed name: | Pantoprazol HEXAL® 40 mg Tabletten |
| --- | --- |
| INN: | Pantoprazole |
| ATC code, if officially registered: | A02BC02 |
| Manufacturer: | Hexal AG, Industriestraße 25, 83607 Holzkirchen |
| Pharmaceutical formulation: | Tablet |
| Dosage: | 40 mg  No dose adjustments are planned. |
| Mode of administration: | Oral before a meal. |
| Storage instructions: | No specific storage instructions |

- - 1. Pharmacological properties

Pantoprazole is an inhibitor of the gastric H^+^/K^+^-ATPase. It is indicated for the treatment of reflux esophagitis, gastric ulcer, and Zollinger-Ellison syndrome.

Pantoprazole is rapidly absorbed; C_max_ is reached 2.5 h after ingestion. Absolute oral bioavailability is about 77 %. There is no influence of food intake on bioavailability. About 98 % of circulating pantoprazol is bound by plasma proteins. The volume of distribution is about 0.15 l/kg. Pantoprazole is primarily metabolized by CYP2C19, to a lesser extent by CYP3A4. The metabolites are excreted mainly by the kidneys (approx. 80 %). In patients with CYP2C19 deficiency (“poor metabolizers”), maximal plasma concentration is increased by about 60 %. Terminal elimination half-time is about 1 h with a clearance of about 0.1 l / h / kg. The elimination does not correlate with the duration of efficacy, which is determined by the irreversible binding of pantoprazole to the gastric parietal cells. [6]

- - 1. Packaging, labelling, and supply

The trial medication is a marketed product. There will be no trial-specific labelling for pantoprazole because this drug is approved for human use in Germany and will be used in unchanged marketed form.

The investigator will confirm correct receipt of the trial medication in writing and ensure that the medication is safely and correctly stored. The investigator will document the use of the trial medication. The site monitor will periodically check the supplies of trial medication held by the investigator to ensure the correct accountability of all trial medication used. At EOT, all unused trial medication will be completely will be completely destroyed or may be transferred for research use to a research facility, both options will be documented. It will be assured that a final report of the drug accountability is prepared and maintained by the investigator.

- - 1. Administration of trial medication

Participants will receive 40 mg of pantoprazole, which will be taken from day 1-9. On V1/day 1 pantoprazole will be taken after PK sampling. Pantoprazole will be taken 1 h before a meal, usually breakfast. On V2/day 6, pantoprazole will be taken 1 h before breakfast and HCQtogether with breakfast. On day 2-5, and 7-9 pantoprazole must be taken at home 1 h before breakfast. No dose adjustments are planned.

- - 1. Adherence and drug accountability

On V1 and V2, drugs will be administered by the investigator. On all other days, the participants are responsible for correct administration; intake will be documented in a diary. A drug accountability log will document the use of drug throughout the study.

- - 1. Safety and tolerability

After oral administration of pantoprazole, the following AEs are known [6]:

Table 5: Pantoprazole adverse drug reactions after oral administration of therapeutic doses [6]:

| Blood und lymphatic system | Agranulocytosis****. Pancytopenia, thrombocytopenia, leukopenia (all*****). |
| --- | --- |
| Immune system | Hypersensitivity (including anaphylaxia)**** |
| Endocrine disorders | Hyperlipidaemia****. Hyponatremia, hypomagnesemia, hypocalcemia, hypokalemia (all******). |
| Psychiatric disorders | Sleep disturbances***, depression****, disorientation*****. Hallucinations and confusion (all******). |
| Nervous system disorders | Headache***, dizziness***, disturbance/loss of taste****, paraesthesia******. |
| Eye disorders | Blurred vision****. |
| Gastrointestinal disorders | Benign fundus polyps**. Diarrhoea, nausea, vomiting, flatulence, obstipation, dry mouth, abdominal pain (all***). |
| Hepatobiliary disorders | Elevated liver enzymes***, elevated bilirubin****. Liver cell damage, jaundice, liver failure (all******). |
| Skin and subcutaneous tissue disorders | Rash***, exanthema***, eruption***, pruritus***, urticaria****, angiooedema****.  Stevens-Johnson syndrome, Lyell syndrome, erythma multiforme, photosensibility, subacute cutaneous lupus erythemasus (all******). |
| Musculoskeletal and connective tissue disorders | Fracture of hip bone, wrist or spine***, arthralgia****, myalgia****, muscle spasm (due to electrolyte disturbances)******. |
| Renal and urinary disorders | Interstitial nephritis******. |
| Reproductive organs | Gynecomastia**** |
| General complaints and symptoms | Asthenia, tiredness, and discomfort (all***).  Elevated temperature****, peripheral oedema****. |

Frequency: *very common **common ≥ 1/100 to < 1/10, ***uncommon ≥ 1/1000 to < 1/100; **** rare ≥ 1/10.000 to < 1/1.000; *****very rare < 1/10.000; ******not known

- - 1. Contraindications

Pantoprazole must not be taken in case of

- Hypersensitivity to the active substance or to any of the excipients.
  - 1. Pregnancy precaution

There are sparse data in humans, which do not show an elevated risk of fetal or neonatal toxicity for pantoprazole. However animal data showed an elevated risk for reproductive toxicity. Pantoprazole did not have any influence on fertility in animal studies. Altogether pantoprazole is not recommended to be used in pregnant women. In a clinical trial pantoprazole would therefore require adequate contraception with a highly effective measure (women) and an effective measure (men) for the duration of the study and at least 1 day afterwards. The pregnancy precaution in this trial, however, will be defined by the IMP which requires the longest pregnancy precautions, which is HCQ.

## Midazolam

Administration of 30 µg p.o. in 100 ml water on V1 and V2.

A pharmacologically inactive dose (30 µg) of Dormicum® will be administered orally as a diagnostic substance to phenotype CYP3A activity using a standard procedure. This dose is > 100 times lower than the regular doses used in a therapeutic setting and will not exhibit any pharmacological activity. Therefore, in this microdose regimen, no benzodiazepine (side) effects are expected; nevertheless, allergic reactions cannot be completely ruled out.

**Table 6**: midazolam dug information

| Marketed name: | Dormicum® V 5 mg / 5 ml |
| --- | --- |
| INN: | Midazolam |
| ATC code, if officially registered: | N05CD08 |
| Manufacturer: | CHEPLAPHARM Arzneimittel GmbH, Ziegelhof 24, 17489 Greifswald |
| Pharmaceutical formulation: | Solution |
| Dosage: | 30 μg |
| Mode of administration: | Oral  0.03 ml of Dormicum® will be administered in 100 ml of water. |
| Storage instructions: | Must not be stored above 30 °C and must be protected from light by keeping the vials in the box until use. |

- - 1. Pharmacological properties

Midazolam, a short-acting benzodiazepine, is a lipophilic substance belonging to the group of imidazo-benzodiazepines. It is mainly used as a sedative, e.g. for premedication or intravenous induction of anaesthesia, and for the treatment of epileptic seizures because of its anxiolytic, hypnotic, anticonvulsive, and muscle relaxant effects. By binding to the GABA-A-receptor, benzodiazepines such as midazolam enhance the affinity of the ligand GABA (ϒ-amino-butyric-acid) leading to increased chloride permeability. Midazolam is selectively metabolized by CYP3A and an established marker for CYP3A activity in clinical pharmacology. In our study, midazolam will be administered p.o. [7]. Midazolam is rapidly absorbed, reaching C_max_ within 60 min in adults under fasting conditions 1 h before food intake, while the rate of absorption 1 h after food intake is delayed and reduced reaching C_max_ only after 115 min. Midazolam is highly lipophilic and distributes extensively.

- - 1. Packaging, labelling, and supply

The trial medication is a marketed product. There will be no trial-specific labelling because this drug is approved for human use in Germany.

The investigator will confirm correct receipt of the trial medication in writing and ensure that the medication is safely and correctly stored. The investigator will document the use of the trial medication. The site monitor will periodically check the supplies of trial medication held by the investigator to ensure the correct accountability of all trial medication used. At EOT, all unused trial medication will be completely destroyed or may be transferred for research use to a research facility, both options will be documented. It will be assured that a final report of the drug accountability is prepared and maintained by the investigator.

- - 1. Administration of trial medication

Participants will receive 0.03 ml of Dormicum® in approximately 100 ml of drinking water once at V1 and V2. The administration of the trial drug will take place at the trial site, 3 h after a meal together with 50 μg yohimbine. No dose adjustments are planned.

- - 1. Adherence and drug accountability

Administration is carried out at the trial site; therefore, a taking and timing adherence of 100 % is expected. A drug accountability log will document the use of drug.

- - 1. Safety and tolerability

Table 7: Midazolam adverse events after oral administration of therapeutic doses [16]

| System Organ Class | Adverse events – frequencies in symbols (Ref. **Fehler! Verweisquelle konnte nicht gefunden werden.**) |
| --- | --- |
| Immune system disorders | *?: Hypersensitivity reactions, angioedema, anaphylactic reaction |
| Psychiatric disorders | *?: Confusion, euphoric mood, hallucinations,: agitation^A^, hyperactivity^A^, hostility^A^, rage reaction^A^, aggressiveness^A^, paroxysmal excitement^A^, dependency, withdrawal syndrome (incl. concomitant convulsions) |
| Nervous system disorders | *?: Prolonged sedation, involuntary movements (incl. tonic-clonic movements, tremor)*, decreased alertness, somnolence, headache, dizziness, ataxia, anterograde amnesia**, convulsions*, prolonged time for reaction |
| Eye disorders | *?: Blurred vision, diplopia, nystagmus, focusing difficulties, cyclic movement of eyelids. |
| Cardiac disorders | *?: Bradycardia, cardiac arrest |
| Vascular disorders | *?: Hypotension, vasodilation, thrombophlebitis, thrombosis |
| Respiratory, thoracic and mediastinal disorders | *?: Respiratory depression, apnea, breathing arrest, dyspnea, spasmodic laryngitis, hiccup |
| Gastro-intestinal disorders | *?: Nausea, vomiting, constipation, dry mouth. |
| Skin and subcutaneous tissue disorders: | *?: Skin rash, urticaria, pruritus. |
| General disorders and administration site conditions | *?: Fatigue, alterations of libido, increased risk of falling in elderly, erythema and pain at the injection site |

^A^: Paradoxical reactions were especially observed in children and/or older patients, *?: no AE frequencies are known for midazolam microdoses

In a therapeutic dosing regimen, AEs are likely. Because this trial uses a microdose setting, administering a single dose of midazolam, which is 100 times lower than the therapeutic dose and thus judged as a biologically inactive dose, AEs are extremely unlikely.

- - 1. Contraindications

Although it is a biologically inactive dose and no other contraindication applies, midazolam must not be administered to patients with known hypersensitivity to midazolam, the brand’s additives, or related substances.

- - 1. Pregnancy precaution

There is no published data about midazolam exposure in the first trimester. However data from diazepam suggested an increased risk for cleft lip-jaws-palates. Other clinical data did not confirm an increased risk. The manufacturer does not recommend the use of the drug in pregnancy unless there is an unequivocal need due to a maternal indication.

Because midazolam will be administered in a microdose setting, no specific pregnancy precautions will be required. The pregnancy precaution in this trial, will anyway be defined by the IMP which requires the highest pregnancy precautions, which is HCQ.

## Yohimbine

Administration of 50 µg p.o. (2 tablets) together with 100 ml water on V1 and V2.

A pharmacologically inactive dose of Yohimbinum hydrochloricum D4® (50 µg) will be administered orally as diagnostic substance to phenotype the CYP2D6 activity. This dose is > 100 times lower than the regular doses used in a therapeutic setting. This dose does not exhibit any pharmacological activity and, therefore, no AEs are expected, nevertheless, allergic reactions cannot be completely ruled out.

**Table 8**: Yohimbine dug information

| Marketed name: | Yohimbinum hydrochloricum D4® |
| --- | --- |
| INN: | Yohimbinum hydrochloricum |
| ATC code, if officially registered: | V60AA |
| Manufacturer: | Deutsche Homöopathie-Union-Arzneimittel GmbH & Co. KG, 76202 Karlsruhe |
| Pharmaceutical formulation: | Tablet |
| Dosage: | 2 x 25 μg |
| Mode of administration: | Oral |

- - 1. Pharmacological properties

Yohimbine is a competitive α2-receptor antagonist, affecting dopaminergic, serotoninergic, and cholinergic neurotransmitters. The definite mode of action for erectile dysfunction is not identified yet. It is postulated that a central nervous pathway leads to raised sexual desire and erection caused by impact on the autonomic nervous system. Furthermore yohimbine possibly causes a dilatation of penile blood vessels. Psychoanaleptic effects have been described as anxiety.

- - 1. Packaging, labelling, and supply

The trial medication is a marketed product. There will be no trial-specific labelling because this drug is approved for human use in Germany and will be used in unchanged marketed form.

The investigator will confirm correct receipt of the trial medication in writing and ensure that the medication is safely and correctly stored. The investigator will document the use of the trial medication. The site monitor will periodically check the supplies of trial medication held by the investigator to ensure the correct accountability of all trial medication used. At EOT, all unused trial medication will be completely destroyed or may be transferred for research use to a research facility, both options will be documented. It will be assured that a final report of the drug accountability is prepared and maintained by the investigator.

- - 1. Administration of trial medication

Participants will receive 2 tablets a 25 μg each as a single oral dose on visit 1 and visit 2. The administration of the trial drug will take place at the trial site, 3 h after a meal together with 0.03 ml of Dormicum®. No dose adjustments are planned.

- - 1. Adherence and drug accountability

On V1 and V2, drugs will be administered by the investigator; therefore, a taking and timing adherence of 100 % is expected. A drug accountability log will document the use of drug.

- - 1. Safety and tolerability

Table 9: Yohimbine adverse events after oral administration of therapeutic doses [17].

| Blood and lymphatic system | Agranulocytosis# |
| --- | --- |
| Immune system | Allergic reaction* |
| Nervous system | Headache**, dizziness*, paraesthesia*, tremor‡ |
| Cardiac and vascular system | Increase of blood pressure and heart rate*, tachycardia*, palpitation*, hypotension* |
| Respiratory, thoracic and mediastinal system | Bronchospasm‡ |
| Gastrointestinal system | Nausea**, vomiting*, inappetence*, epigastric pain*, diarrhoea* |
| Dermis | Skin flushing*, rash*, urticarial*, increased growth of hair*, exanthema^‡^ |
| Renal and urinary system | Increased urinary urgency*, dysuria^‡^, decreased urinary urgency^‡^, genital pain^‡^ |
| General | Sweating*, chills* |
| Psychiatric disorders | Agitation**, irritability**, sleeplessness**, nervousness* |

Frequency: ***very common (≥ 1/10), **common (≥ 1/100 to < 1/10), *uncommon (≥ 1/1,000 to < 1/100), †rare (≥ 1/10,000 to < 1/1,000), ‡very rare (< 1/10,000), #not known (cannot be estimated from the available data)

Because this trial uses a microdose setting, administering a single dose of 50 µg yohimbine is judged as a biologically inactive dose, encountering AEs is extremely unlikely.

- - 1. Contraindications

Although it is a biologically inactive dose and other contraindications do not apply, yohimbine must not be administered to patients with known hypersensitivity to yohimbine or to any of the excipients. Yohimbinum hydrochloriucm D 4® also contains wheat starch, lactose, and magnesium stearate.

- - 1. Pregnancy precaution

In animal studies to evaluate genotoxicity and effects on fertility in male rats there were no pathologic findings. There are no preclinical data on prenatal toxicity available for yohimbine [18].

Because yohimbine will be administered in a homeopathic microdose setting, no specific pregnancy precautions will be required. The pregnancy precaution in this trial, will anyway be defined by the IMP which requires the highest pregnancy precautions, which is HCQ.

# Assignment of participant codes

After signing the informed consent form, every potential volunteer receives a screening number at the SCR visit. When the participant is randomized to the trial, he/she will be given a consecutive subject number. Participants withdrawn from the trial retain their subject number. New participants must always be allocated a new screening and subject number.

## Blinding and unblinding

This is an open trial; there will be no blinding.

## Randomization

A randomization list will be created. Participants will be assigned random numbers based on consecutive enrolment.

# Monitoring

Qualified personnel from the Department of Clinical Pharmacology and Pharmacoepidemiology will carry out the clinical monitoring. The monitor is responsible for checking the quality of data and adherence to the trial protocol and to legal and ethical requirements according to local laws and the principles of GCP. Source data verification is an essential part of the monitoring process and the investigator must grant direct access to all data.

The monitor will work according to the pertinent versions of the monitoring SOPs of the Department of Clinical Pharmacology and Pharmacoepidemiology.

The following variables will be verified to an extent that will be defined in a monitoring plan prior to trial start:

- Informed consent,
- Inclusion/exclusion criteria,
- Demographic data (identity number, age, and sex),
- Drug accountability, handling, and storage,
- AEs and especially SAEs,
- Premature trial termination for individual participants (dropouts and withdrawals), and
- Adherence to the protocol and to GCP standards.

# End of trial

## Regular trial end

The end of trial is defined as the date of database closure.

## Premature end of trial

The responsible EC and the competent authorities must be informed about any premature closure of the trial. Furthermore, the EC of the Medical Faculty of Heidelberg University and the competent authority themselves may decide to stop or suspend the trial.

All involved investigators have to be informed immediately about the cessation/suspension of the trial. The decision is binding to all investigators.

- - 1. Early termination of the trial

If new information on the risk-to-benefit ratio of the drug or on the treatment methods used in the trial is coming to light and safety concerns arise, the sponsor reserves the right to interrupt or terminate the project. In addition the principal investigator (PI) may decide at any time to terminate the trial due to a situation where an unexpected high frequency of AEs will occur or due to financial reasons.

- - 1. Stopping criteria for the trial

The study must be stopped if more than one serious or grade IV related AE will have occurred. SAEs definitely caused by accidental injuries will not be counted towards the stopping criteria. Laboratory abnormalities without any clinical symptoms will not count as stopping criteria.

## Withdrawal of participants

At any time, a participant may withdraw his/her consent for further participation. In this case, the investigator will have to exclude the participant from the trial. The participant is not required to state a reason for withdrawal.

The investigator may decide to withdraw a participant, if the participant suffers from a SAE regardless of whether it is drug-related or not, in case of a major protocol violation by the participant, or if another event occurs whose interference with the investigation of the target criteria is suspected or considered critical.

## Dropouts

Dropouts in the SCR period will be replaced by rescreening, participants may be rescreened more than once if the dropout was for administrative reasons or intercurrent disease. Dropouts after trial medication intake cannot be rescreened.

# Data analysis

## Sample size and power calculation

The planned sample size is 24 healthy volunteers, 12 in each arm.

The sample size calculation is based on previous data after single doses of HCQ [1]. An online calculator was used for the sample size calculation (http://hedwig.mgh.harvard.edu/sample_size/js/js_parallel_quant.html). With the AUC_0-72 h_ data available, a sample size of 14 (7 in each group) is needed to detect a 30 % difference in AUC_0-72 h_ with a power of 0.8 and an α-error of 5 %. Therefore, a sample size of 12 per group is regarded as sufficient to detect a PPI-induced difference on HCQ AUC_0-72 h_ exceeding the bioequivalence range (80-125 %).

Up to 12 volunteers may be replaced if enrolled volunteers drop out prior to exposure with HCQ or prior to completing the 72 h PK sampling period.

## Statistical analysis plan

- - 1. Analysis sets

All volunteers who received trial treatment will be analysed. Analysis of the primary endpoint will be done in the complete case set. Safety analyses will be done with all volunteers who received trial medication.

- - 1. PK parameters

Standard PK parameters will be calculated using the pertinent version of Kinetica (Thermo Fisher Scientific, Waltham, USA). Graphical analyses will be done using the pertinent version of Prism (GraphPad Software Inc., La Jolla, USA). Equivalent software for the dedicated use of PK analysis or graphical and statistical analysis may be used if the SOP of the trial unit may specify this at a later point.

- - 1. Statistical analysis

The primary outcome measures are the difference in AUC_0-72 h_ and C_max_ of a single dose of 400 mg oral HCQ with or without concomitant pantoprazole therapy.

The following parameters will be reported through detailed descriptive statistics and graphics and, if applicable, changes will be analysed using ANOVA or paired t-tests at an α = 5 % level:

- AUC_2-4 h_, AUC_0-6 h_ and C_max_ of microdosed midazolam and microdosed yohimbine,
- Correlation of concentrations of HCQ in whole blood as compared to concentrations in plasma and PBMCs.
- AUC_0-72 h_ and C_max_ of desethyl-HCQ with and without pantoprazole.
- Metabolic rations will be analysed where metabolites are available

Seriousness, severity, relationship, and frequency of AEs during the treatment period will be tabulated and summarized.

Further descriptive or non-confirmatory statistical analyses may be done at the discretion of the investigator.

- - 1. Procedure for handling missing, unused, and inconsistent data

Individual missing or inconsistent data will be subject to a simple query edit process. Eventually missing data will not be imputed.

# Ethic and legal aspects

## General

The trial will be carried out in accordance with the pertinent versions of the legal requirements as laid down in the German ‘Arzneimittelgesetz’ (AMG), GCP-V, the professional law (Berufsordnung für Ärzte in Baden-Württemberg), the applicable version of the Declaration of Helsinki first adopted by the World Medical Association in June 1964, and the ICH recommendations on GCP. The State Data Protection Law (Landesdatenschutzgesetz Baden Württemberg) and the General Data Protection Regulation of the EU (Datenschutzgrundverordnung, DSGVO, EU 2016/679) will also be applied.

## Financial disclosure of investigator

Before the start of the trial, the investigator will disclose to the sponsor any proprietary or financial interests he or she might hold in the sponsor company, in the IMPs, or any commercial organization being involved in the clinical trial. The investigator has also to confirm that he/she has not entered into any financial arrangement, whereby the value of compensation paid could affect the outcome of the clinical trial. The investigator agrees to update this information in case of significant changes.

## Submission to the Ethics Committee and Competent Authority (BfArM)

The investigator submits the required documents to the responsible EC of the Medical Faculty of Heidelberg University and obtains the opinion of the Committee in writing. The investigator also submits the required documents to the Bundesinstitut für Arzneimittel und Medizinprodukte (Federal Institute for Drugs and Medical Devices) (BfArM) and obtains the approval in writing. Participants will not be included until unconditional approval of BfArM and EC has been received. EC and BfArM will be informed of all SAE (also including SUSARs) likely to affect the safety of the participants or compromise the continuation of the trial.

## Notification of authorities

According to § 67 of the German ´Arzneimittelgesetz´, the ´Regierungspräsidium Karlsruhe´ and the competent authority (BfArM) will be notified about the start and the end of the trial. The competent authority will be notified about substantial amendments.

A Development Safety Update Report will be prepared and sent to the EC and to BfArM once per year, if the trial duration is equal or longer than one year.

A final report will be issued to the national competent authority (BfArM) according to § 42b AMG in the format of the synopsis of ICH Topic E3 – Note for Guidance on Structure and Content of Clinical Study Reports within a year after the end of the trial. The trial results will also be published in the EudraCT clinical trial database.

## Responsibilities

The investigator ensures that all team members are informed adequately about the protocol, all amendments to the protocol, the study procedures, and study specific duties and tasks. The investigator will maintain a list to delegate tasks to the team members. Further details are mentioned in Appendix 16.2.

## Insurance

According to § 40 AMG, the sponsor has to subscribe to an insurance policy covering, in its terms and provisions, its legal liability for injuries caused to participating persons and arising out of this research performed strictly in accordance with the scientific protocol as well as with applicable law and professional standards. The insurance also includes the coverage of travel accidents (travel accident insurance). The insurance was taken out at

HDI Gerling Versicherung AG

Am Schönenkamp 45

40599 Düsseldorf

(insurance number: 57 010310 03018//03152).

Any impairment of health that might have occurred in consequence of trial participation must be notified to the insurance company. The participant is responsible for notification. The insured person will agree with all appropriate measures serving for clarification of the cause and the extent of damage as well as the reduction of damage.

During the conduct of the trial, the participant must not undergo other clinical treatment except for cases of emergency. The subject is bound to inform the investigator immediately about any AEs and additionally taken drugs. The terms and conditions of the insurance should be delivered to the participant.

The insurance company has to be informed about all amendments that could affect participants’ safety.

## Confidentiality

The data obtained in the course of the trial will be treated pursuant to the State Data Protection Law (Landesdatenschutzgesetz Baden Württemberg) and the General Data Protection Regulation of the EU (DSGVO, EU 2016/679).

During the clinical trial, participants’ identification and results will be documented in a file comparable to a patient file that follows all rules of confidentiality of the Heidelberg University Hospital.

Relevant data for the trial will be documented in a database in a pseudonymized way in order to prevent any possibility for third parties to identify the participant. Also if data are transferred to third parties or published, they will only be used in the pseudonymized way. Trial findings stored on a computer will be stored in accordance with the data protection law and will be handled in strictest confidence. For protection of this data, organisational procedures are implemented to prevent distribution of data to unauthorised persons. The appropriate regulations of local data will be fulfilled in their entirety.

The participant consents in writing to release the investigator from his professional discretion in so far as to allow inspection of original data for monitoring purposes by health authorities and authorised persons. Authorised persons (clinical monitors, inspectors, and auditors) may take insight into personal participant-related data collected during the trial ensuring the data protection law.

The investigator will maintain a participant identification list (participant numbers with the corresponding names) to enable records to be identified.

Volunteers who did not consent to circulate their pseudonymised data will not be included into the trial.

## Archiving

The investigator is responsible for archiving the participants’ files and also all documents concerning the trial for at least 10 y after the end of the trial. These personalized data will be destroyed at the latest after 15 y.

## Protocol modifications and amendments

If the trial protocol has to be changed substantially after approval, a written amendment is required that must be signed by the same persons as mentioned in the trial protocol. Any protocol amendment will only be implemented after approval has been granted by the EC and the competent authorities. Any substantial protocol amendment affecting the benefit-to-risk ratio must be approved by the responsible EC and BfArM and must be notified to the local regulatory authority (Regierungspräsidium Karlsruhe). These procedures must be completed before any modifications can come into operation, except when they are necessary to eliminate immediate hazards for the trial participants. Participants will be informed about relevant changes in the trial and will be asked to re-consent in writing

## Access to source data, quality control, and data handling

For the purpose of ensuring compliance with the clinical trial protocol, GCP, and applicable regulatory requirements, the investigator should permit auditing/inspection/monitoring by responsible regulatory authorities. The investigator agrees to allow the auditors/inspectors/monitors to have direct access to the trial records for review, being understood that this personnel is bound by professional secrecy, and as such will not disclose any personal identity or personal medical information. The investigator will make every effort to help with the performance of the audits and inspections, giving access to all necessary facilities, data, and documents. The confidentiality of the data should be verified, and the protection of the participants should be respected during these inspections.

## Participant information and informed consent

Before being admitted to the clinical trial, the participant must consent to participate after being fully informed by the investigator or a designated member of the investigating team about the nature, importance, risks and individual consequences of the clinical trial and their right to terminate the participation at any time. The participant should also have the opportunity to consult the investigator, or a physician member of the investigating team about the details of the clinical trial. The informed consent to participate in the clinical trial may be withdrawn by the participant verbally in the presence of, or in written form directed to, the investigator or a physician member of the investigating team at any time during the trial. The participant must not entail any disadvantage therefore or be coerced or unduly influenced to continue to participate. Furthermore, the participant is not obligated to disclose reasons for the withdrawal of the consent.

After reading the informed consent document, the participant must give consent in writing. The participants consent must be confirmed by the personally dated signature of the participant and by the personally dated signature of the physician conducting the informed consent discussion. A copy of the signed informed consent document must be given to the participant. The documents must be in a language understandable to the participant and must specify who informed the participant.

If the participant is unable to write, oral presentation and explanation of the content of the informed consent form and of the data protection information must take place in the presence of an impartial witness. The witness and the physician conducting the informed consent discussions must also sign and personally date the consent document. The witness must not be in any way dependent on the sponsor of the trial, the trial site or any member of the investigating team (e.g. an employee at the trial site).

The participants will be informed as soon as possible if new information may influence his/her decision to participate in the trial. The communication of this information should be documented.

## List of participants (participants’ log)

The investigator keeps a confidential list with full names, dates of birth, and addresses of all individuals participating in the trial, giving reference to the participants’ records. For enrolled participants, the date of enrolment and the allocation/random number must be recorded. With this list, it must be possible to identify the participants and their medical records, e.g. in case of questions arising after completion of the trial. Care must be taken that this list is kept confidential by the investigator. The identity of the participants must not be revealed to unauthorised persons.

## Participant medical records / source data

Participant medical records must be kept for all volunteers taking part in the trial. Participation in the trial, dates of participant information and signed informed consent, administration of trial medication, and key data recorded during the trial must be documented in the participant’s medical record as source data unless specified otherwise in this trial protocol. Data in the participant’s medical record (including original laboratory reports etc.) are the ‘source data’ and entries in the CRF have to be checked against these data (source data verification) by the monitor.

## Data management

All data required by the protocol will be recorded on source data. The data will timely be entered in a database via an electronic CRF (eCRF), according to the trial site’s SOP. The software ensures that the participants´ pseudonymity will be maintained. All data to be recorded according to this trial protocol must be documented in the eCRF. After checking for plausibility, consistency, and completeness, queries will be edited and resolved. Changes in the eCRF are traceable by an audit trail.

After all data are entered into the database and the queries are closed the investigator and deputy will agree on the database closure.

## Financing

The trial will be fully financed by the Department of Clinical Pharmacology and Pharmaco­epidemiology of Heidelberg University.

## Publication of trial results

The results of this investigation will be published in an international scientific peer-reviewed journal.

# References

1. Collins, K.P., K.M. Jackson, and D.L. Gustafson, *Hydroxychloroquine: A Physiologically-Based Pharmacokinetic Model in the Context of Cancer-Related Autophagy Modulation.* J Pharmacol Exp Ther, 2018. **365**(3): p. 447-459.

2. Tett, S., et al., *Insights from pharmacokinetic and pharmacodynamic studies of hydroxychloroquine.* Agents Actions Suppl, 1993. **44**: p. 145-90.

3. Munster, T., et al., *Hydroxychloroquine concentration-response relationships in patients with rheumatoid arthritis.* Arthritis Rheum, 2002. **46**(6): p. 1460-9.

4. Arnaud, L., et al., *The importance of assessing medication exposure to the definition of refractory disease in systemic lupus erythematosus.* Autoimmun Rev, 2011. **10**(11): p. 674-8.

5. Sanofi-Aventis, *Fachinformation Quensyl.* Februar 2018.

6. Hexal, *Fachinformation Pantoprazol Hexal 40 mg.* November 2018.

7. Halama, B., et al., *A nanogram dose of the CYP3A probe substrate midazolam to evaluate drug interactions.* Clin Pharmacol Ther, 2013. **93**(6): p. 564-71.

8. Hohmann, N., et al., *Midazolam microdose to determine systemic and pre-systemic metabolic CYP3A activity in humans.* Br J Clin Pharmacol, 2015. **79**(2): p. 278-85.

9. Vay, M., et al., *Oral Yohimbine as a New Probe Drug to Predict CYP2D6 Activity: Results of a Fixed-Sequence Phase I Trial.* Clin Pharmacokinet, 2020, epub ahead of print

10. Yao, X., et al., *In Vitro Antiviral Activity and Projection of Optimized Dosing Design of Hydroxychloroquine for the Treatment of Severe Acute Respiratory Syndrome Coronavirus 2 (SARS-CoV-2).* Clin Infect Dis, 2020, epub ahead of print.

11. Burhenne, J., et al., *Quantification of femtomolar concentrations of the CYP3A substrate midazolam and its main metabolite 1'-hydroxymidazolam in human plasma using ultra performance liquid chromatography coupled to tandem mass spectrometry.* Anal Bioanal Chem, 2012. **402**(7): p. 2439-50.

12. Vay, M., et al., *Quantification of microdosed oral yohimbine and its major metabolite in human plasma in the picogram range.* Bioanalysis, 2019. **11**(16): p. 1459-1467.

13. US Department of Health and Human Services. Guidance for industry, bioanalytical method validation- Draft Version. www.fda.gov/downloads/Drugs/GuidanceComplianceRegulatoryInformation/Guidances/ucm070107.pdf> (2013). Accessed 09 April 2020..

14. European Medicines Agency. Guideline on bioanalytical method validation. www.ema.europa.eu/docs/en_GB/document_library/Scientific_guideline/2011/08/WC500109686.pdf> (2012). Accessed 09 April 2020..

15. Carmichael, S.J., B. Charles, and S.E. Tett, *Population pharmacokinetics of hydroxychloroquine in patients with rheumatoid arthritis.* Ther Drug Monit, 2003. **25**(6): p. 671-81.

16. *CHEPLAPHARM Arzneimittel GmbH, Dormicum® V 5 mg / 5 ml, October 2019.*

17. Deutsche Homöopathie-Union,*Gebrauchsinformation Yohimbinum hydrochloricum D 4*, undated.

18. Cheplapharm, *Fachinformation* *YOCON-GLENWOOD 5mg Tabl (Yohimbine).* February 2017.

# Appendix

## Definition of AEs

**Definition of an AE**

An AE is any unfavourable and unintended sign (including an abnormal laboratory finding), symptom, or disease temporally associated with the use of a medical treatment or procedure that may or may not be considered related to the medical treatment or procedure. Every change in laboratory values of vital function of the participant that leads to discontinuation of the trial medication or discontinuation of the trial should be regarded as AE.

**Definition of a SAE**

A SAE is an AE that at any time during the period of observation:

- results in death,
- is immediately life-threatening,
- requires or prolongs hospitalisation,
- results in persistent or significant disability or incapacity,
- involves congenital anomaly,
- is medically important, or
- requires medical intervention to prevent permanent impairment or damage.

**Definition of unexpected AE**

An unexpected AE is an AE of which the nature and intensity is not consistent with the applicable product information.

**Definition of suspected unexpected serious adverse reaction (SUSAR)**

A SUSAR is a suspected unexpected serious adverse reaction. All AE that are suspected to be related to an IMP and that are both unexpected and serious are considered to be SUSARs.

**Intensity**

The assessment of intensity will be based on the investigator’s clinical judgment guided by the pertinent version of the Common Terminology Criteria for AEs (CTCAE) using the following definitions:

Mild: an event that is easily tolerated by the participant, causing minimal discomfort and not interfering with activities of daily life: asymptomatic or mild symptoms; clinical or diagnostic observations only; not requiring intervention.

Moderate: an event that is sufficiently discomforting to interfere with normal activities of daily life: minimal, local or non-invasive intervention indicated.

Severe: An event that prevents normal activities of daily life: Severe or medically significant but not immediately life-threatening; hospitalization or prolongation of hospitalization indicated.

Life-threatening: Life-threatening consequences, urgent intervention indicated.

Death: Death related to AE.

**Causality**

The causal relationship of an AE to treatment (treatment indicates all IMPs including placebo) has to be assessed in accordance with WHO criteria in a modified version: Edwards IR, Biriell C. Harmonisation in pharmacovigilance. Drug Saf 2004;10:93-102.

The investigator will use clinical judgment to determine the relationship. Alternative causes, such as natural history of the underlying diseases, concomitant therapy, other risk factors etc. will be considered. The investigator will also consult the summary of product characteristics. The relationship between the drug and the occurrence of each AE will be evaluated and classified as:

Certain: A clinical event, including laboratory test abnormality, occurs in a plausible time relationship to drug administration, and cannot be explained by concurrent disease or other drugs or chemicals. The response to withdrawal of the drug (dechallenge) should be clinically plausible. The event must be definitive pharmacologically or phenomenologically, using a satisfactory rechallenge procedure if necessary.

Probable: A clinical event, including laboratory test abnormality, with a reasonable time sequence to administration of the drug, unlikely to be attributed to concurrent disease or other drugs or chemicals, and which follows a clinically reasonable response on withdrawal (dechallenge). Rechallenge information is not required to fulfil this definition.

Possible: A clinical event, including laboratory test abnormality, with a reasonable time sequence to administration of the drug, but which could also be explained by concurrent disease or other drugs or chemicals. Information on drug withdrawal may be lacking or unclear.

Unlikely: A clinical event, including laboratory test abnormality, with a temporal relationship to drug administration which makes a causal relationship improbable, and in which other drugs, chemicals, or underlying disease provide plausible explanations.

Unclassified: A clinical event, including laboratory test abnormality, reported as an adverse reaction, about which more data are essential for a proper assessment or the additional data are under examination.

Unclassifiable: A report suggesting an adverse reaction which cannot be judged because information is insufficient or contradictory, and which cannot be supplemented or verified.

Not related: A clinical event, including laboratory test abnormality, with no relationship to drug administration and in which other drugs, chemicals, or circumstances provide plausible explanations.

**Reporting of SAE by the investigator**

All SAEs must be reported by the investigator to the responsible Safety Officer within 24 h after the SAE becomes known using the “SAE” form. The initial report must be as complete as possible including details of the current illness and (serious) AE and an assessment of the causal relationship between the event and the trial medication. (The investigator must also inform the site monitor in all cases).

**SUSAR - Responsibilities of the PI**

The PI is responsible for the notification of the SUSAR to the EC and competent authority of each concerned Member State of findings that could adversely affect the health of the participants, impact on the conduct of the trial, or alter the competent authorisation to continue the trial in accordance with Directive 2001/20/EC.

In the case of SUSARs that resulted in death or were life-threatening the competent authority, the EC, and all investigators should be notified as soon as possible but no later than 7 calendar days after the PI has first knowledge of the minimum criteria for expedited reporting. In each case relevant follow-up information should be sought and a report completed as soon as possible. It should be communicated to the competent authority and the EC in the concerned Member States within an additional eight calendar days. All other SUSARs and safety issues must be reported to the competent authority and the EC in the concerned Member States as soon as possible but no later than 15 calendar days after the PI has first knowledge of the minimum criteria for expedited reporting. Further relevant follow-up information should be given as soon as possible. Person-related data have to be pseudonymised by using a participant code number.

Each SUSAR has to be reported as a single case to the competent authority in the concerned Member State. Expedited reporting is not usually required: for reactions which are serious but expected.

For regulatory purposes, initial expedited reports should be submitted within the time limits as soon as the minimum following criteria are met:

- A suspected IMP
- An identifiable participant (pseudonymised)
- An AE assessed as serious and unexpected, and for which there is a reasonable suspected causal relationship
- An identifiable reporting source
- A unique trial identification number (EudraCT number)
- An assessment of causality, intensity, data on the started therapies, and the outcome.

**Outcome of an AE**

The outcome of an AE at the time of the last observation will be classified as:

‘Recovered/resolved’: All signs and symptoms of an AE disappeared without any sequels at the time of the last interrogation.

‘Recovering/resolving’: The intensity of signs and symptoms has been diminishing and/or their clinical pattern has been changing up to the time of the last interrogation in a way typical for its resolution.

‘Not recovered/not resolved’: Signs and symptoms of an AE are mostly unchanged at the time of the last interrogation.

‘Recovered/resolved with sequel’: Actual signs / symptoms of an AE disappeared but there are sequels related to AE.

‘Fatal’: Resulting in death. If there are more than one AE only the AE leading to death (possibly related) will be characterised as “fatal”.

‘Unknown’: The outcome is unknown or implausible and the information cannot be supplemented or verified.

**Action taken with IMP**

The action taken with IMP will be assigned to one of the following categories:

‘Dose not changed’: no change in the dose of IMP.

‘Dose reduced’: reduction of IMP dose.

‘Dose increased’: increase in IMP dose.

‘Drug withdrawn’: discontinuation of IMP.

‘Unknown’: the information is unknown or implausible and it cannot be supplemented or verified.

‘Not applicable’: No answer possible (e.g. the participant is dead).

‘Countermeasures‘: Refers to the specific actions taken to treat or alleviate AE or to avoid their sequels.

**Countermeasures to AE**

The following categories will be used to categorise the countermeasures to AE:

None: no action taken.

Drug treatment: newly prescribed medication or change in dose of a medication

Others: other countermeasures, e.g. a surgical procedure

**Procedures for reporting of SUSAR**

In accordance with the internal University regulations the PI of an Investigator Initiated Trial also acts as sponsor. All SAEs (including SUSARS) are documented on the international CIOMS form (VA KliPS-5 und SOP KliPS-5-01).

According to § 42 and § 63b AMG (Arzneimittelgesetz/German Drug Law) serious adverse reactions fulfilling specified criteria have to be reported by the PI within 15 d to the EC, the competent authorities of the member states in whose territory the clinical trial is conducted, and to the Investigators participating in the trial. SUSARS that are fatal or life-threatening have to be reported even within 7 d (GCP-V §13).

**References**

- Detailed guidance on the European database of Suspected Unexpected Serious Adverse Reactions (Eudravigilance – Clinical Trial Module) April 2004.
- Verordnung über die Anwendung der Guten Klinischen Praxis bei der Durchführung von klinischen Prüfungen mit Arzneimitteln zur Anwendung am Menschen (GCP Verordnung-GCP-V), Bundesgesetzblatt Jahrgang 2004 Teil I Nr. 42. Zuletzt geändert durch Art. 8 G v. 19.10.2012 I 2192
- Clinical Safety Data Management: Data elements for transmission of individual case safety reports, CPMP/ICH/287/95, The European Agency for the Evaluation of Medicinal products, London, 16. November 2000
- Note for Guidance on Definitions and Standards for expedited reporting (CPMP/ICH/3945/03), The European Agency for the Evaluation of Medicinal products, London, November 2003

## Responsibilities of the PI

During the conduct of the study the PI is responsible for the following points. In accordance with GCP he or she should:

**Before the beginning of the study:**

- Have all study documents concerning the study drugs.
- Ensure that the PI and his team will be available to conduct the study under good conditions.
- Organise the technical facilities at disposition so that the study is carried out correctly.
- Submit the protocol, the compound information sheet, and the information and informed consent form (as applicable) to the EC.
- Ensure that the batches of study drugs are stored under good storage conditions in a specific place inaccessible to third parties.
- Sign to confirm the agreement to carry out the clinical study protocol in compliance with ICH-GCP, GCP-V, and the AMG.
- Supply each participant with the required information concerning the study drug and the study procedure before inclusion into the study.

**During the study**

- Ensure that the protocol and its appendices are faithfully followed. Notably, if treatment is discontinued for any participant, every effort will be made to obtain information about this participant as promptly as possible after discontinuation of treatment.
- Give the participant any new information concerning the study drug which may modify his consent to participate in the study.
- If there is a modification to the risk / benefit ratio, submit these to the EC.
- Guarantee that the drugs will be administered exclusively within the context of the protocol.
- Mark the participants’ files to indicate their participation in a clinical study, showing dates and data involved in the study.
- Promptly establish a SAE report on the prepared SAE form in case of a SAE.
- Document participants included in the study on a continuously updated list.
- Document personnel involved in the study and their delegated duties on a continuously updated list.

**After the study**

- Retain the list of names of participants included in the study (to be attached to the study documentation).
- Ensure that the participants’ files and all documents relating to the study are archived for at least 10 y after the end of the study (the patient identification list has to be archived for at least 15 y)
- Date and sign the final report.
- All corrections or additions to a final report must be made in the form of a justified amendment, signed and dated by the investigator.

## Contraindicated drugs

Use of any medication (prescription medication, non-prescription medication including multivitamin or herbal preparations) with active ingredients except hormonal contraception and thyroid hormones is not allowed during the trial. However, if a medical emergency arises it will be specifically pointed out that the following drugs should not be combined.

HCQ:

- no contraindicated drugs
- use nephrotoxic or hepatotoxic drugs with caution within 5 half-lives/90 days after the intake of HCQ

Pantoprazole

- no contraindicated drugs

Yohimbine and midazolam are only administered as microdosed drugs in this trial without pharmacological activity. Therefore contraindicated drugs are not relevant.
