# Supplementary material for: Impact of pantoprazole on absorption and disposition of hydroxychloroquine, a drug used in Corona Virus Disease-19 (Covid-19): A structured summary of a study protocol for a randomised controlled trial
Source: Trials. 2020 Jun 29;21:584. doi: 10.1186/s13063-020-04476-y (PMC7322698; doi:10.1186/s13063-020-04476-y)
Supplement: Supplementary file 2 — Additional file 2. SPIRIT 2013 Checklist: Recommended items to address in a clinical trial protocol and related documents. [file 13063_2020_4476_MOESM2_ESM.doc]

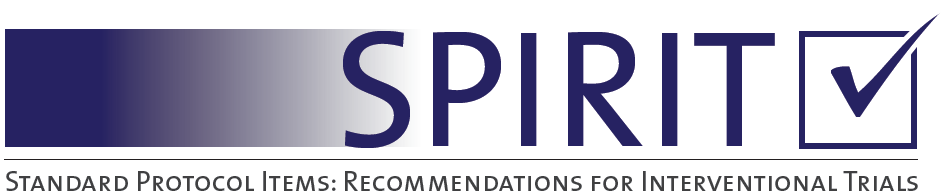


SPIRIT 2013 Checklist: Recommended items to address in a clinical trial protocol and related documents*

| Section/item | ItemNo | Description |
| --- | --- | --- |
| **Administrative information** | | |
| Title | 1 | Impact of pantoprazole [compared to control] on absorption and disposition of hydroxychloroquine, a drug used in Corona Virus Disease-19 (Covid-19) [in healthy volunteers] |
| Trial registration | 2a | EudraCT Number: 2020-001470-30  DRKS and Clinical Trials Registry Platform: DRKS00021573 |
|  | 2b | Please see: World Health Organization Trial Registration Data Set (enclosed) |
| Protocol version | 3 | Version 2.1. dated 24/04/2020 |
| Funding | 4 | Department of Clinical Pharmacology and Pharmacoepidemiology, Heidelberg University Hospital, Germany |
| Roles and responsibilities | 5a | Prof. Dr. med. Walter E. Haefeli, Principal Investigator  Dr. med. Antje Blank, Deputy Investigator  Dr. med. Dipl. Vw. Felicitas Stoll, Deputy Investigator  Prof. Dr. med. Dipl. Phys. Gerd Mikus, Biostatistician  Heidelberg University Hospital  Department of Clinical Pharmacology and Pharmacoepidemiology  Im Neuenheimer Feld 410  69120 Heidelberg  Germany |
|  | 5b | Ruprecht-Karls-University Heidelberg, Medical Faculty  represented in law by Heidelberg University Hospital  and its Commercial Managing Director Katrin Erk  Im Neuenheimer Feld 672  69120 Heidelberg  Germany |
|  | 5c | The legal sponsor of the trial is the Ruprecht-Karls-University Heidelberg, Medical Faculty, represented in law by Heidelberg University Hospital, Germany, and its commercial director Katrin Erk. The trial is investigator-initiated, therefore, the main investigator has obtained all rights and duties from the legal sponsor except contract management. The legal sponsor itself does not have a role in collection, management, analysis, and interpretation of data; writing of the report; and the decision to submit the report for publication. |
|  | 5d | Not applicable |
| Introduction |  |  |
| Background and rationale | 6a | The search for effective therapy of Corona Virus Disease-19 (COVID-19) has put HCQ into the focus of interest. In the absence of proven effective drugs, explorative treatment schedules for COVID-19 patients now foresee the use of oral HCQ although the risk-benefit ratio remains currently unclear. Particularly in seriously ill patients and elderly patients, PPI therapy for prophylaxis or treatment of gastric or duodenal ulcers or gastroesophageal reflux disease is common. Bioavailability of oral HCQ is highly variable and reported values range between 30-70 %. With acid dissociation constant (pKa) values of 9.67 and 8.27, HCQ crosses membranes readily at neutral pH but accumulates in acidic compartments where it is protonated and thus charged which increases solubility. Therefore, changes in gastric pH could be associated with alterations of HCQ bioavailability and exposure because of impaired solubility and this could translate into variability of treatment success.  We hypothesize that absence and presence of PPI could modulate the outcome of HCQ therapy and unintended low HCQ (or active metabolite) blood concentrations could be related to an unfavourable course of disease, as observed in patients with systemic lupus erythematosus. In particular, we expect that in the absence of acid-modifying therapies oral HCQ will be trapped in the acidic environment of the stomach whereas PPI therapy will increase pH and thus render the compound more lipophilic and hence foster absorption. In addition, HCQ could act as a perpetrator and victim at CYP3A, CYP2D6, and possibly also of the CYP2C family.  This trial will investigate the potential effect of pantoprazole on HCQ absorption in vivo. HCQ blood concentrations will be measured in participants under pantoprazole treatment, and will be compared to participants who do not receive PPI or other antacid.  Furthermore, we aim to characterize the relationship between relevant CYP activities metabolic profile of HCQ by administering a microdose of midozolam, and yohimbine. |
|  | 6b | Not applicable. Participants of the control group will not receive any comparator drug. |
| Objectives | 7 | - - 1. Primary objective - Evaluation of the effect of the PPI pantoprazole on the absorption of HCQ in healthy volunteers.   - 1. Secondary objectives - Comparison of HCQ concentrations in whole blood as compared to plasma and intracellular concentration as measured in target cells (peripheral blood mononuclear cells, PBMCs). - Evaluation of HCQ as a perpetrator drug in DDI at the level of CYP3A and CYP2D6   - 1. Exploratory objectives - Evaluation of the effect of pantoprazole on the disposition of major HCQ metabolites |
| Trial design | 8 | Single-centre, open-label, parallel group, two-arm, one-sequence phase I DDI trial in healthy volunteers. |
| Methods: Participants, interventions, and outcomes | | |
| Study setting | 9 | Clinical Pharmacological Trial Center of Heidelberg University Hospital, Germany |
| Eligibility criteria | 10 | Inclusion criteria:   1. Age 18-60 y inclusive at the time of consent, 2. Males and females of child-bearing potential who are willing to use a highly effective method of contraception during the treatment and for 3 months after last administration of the IMP or women not of child-bearing potential (WNCBP) or individuals who are convincingly sexually abstinent. 3. Understanding, ability, and willingness to fully comply with trial interventions and restrictions, 4. Willingness to participate in a genotyping study (K093), and 5. Ability to provide written, personally signed and dated informed consent to participate in the trial, in accordance with the International Conference on Harmonisation (ICH) Good Clinical Practice (GCP) Guideline E6, and applicable regulations, prior to any trial-related interventions.   Exclusion criteria:   1. Clinically significant or relevant abnormalities in the medical history, physical examination, and laboratory evaluation as assessed by the investigator, 2. Any medical disorder that may require treatment or make the participant unlikely to fully complete the trial, or any condition that presents undue risk from the IMPs or trial interventions, 3. Clinically relevant ongoing or clinically relevant history of physical or psychiatric illness as judged by the investigator, especially a) Gastrointestinal, neurological, or haematological diseases b) history of psoriasis, porphyria, or epilepsy 4. Pregnancy or breast feeding, 5. Any acute or chronic illness or clinically relevant finding known or expected to modify absorption, distribution, metabolism, or excretion of HCQ, midazolam, yohimbine, or pantoprazole, 6. Any known history of severe allergic or anaphylactic reactions to drugs or food or any other clinically significant allergies (except mild forms of hay fever), 7. Any known allergies to the compound or further ingredients of HCQ, quinine, pantoprazole, midazolam, or yohimbine preparations, 8. Prolonged QTc time: women: QTcF > 460 ms, men: QTcF > 440 ms 9. Clinically relevant findings in any of the following investigations at SCR. Minor deviations of laboratory values from the normal range can be acceptable, if judged by the investigator to be of no clinical relevance for this trial: 10. Hemoglobin (Hb) < 12 g/dl (males) or < 11 g/dl (females), 11. Creatinine (Crea) clearance (Cl) < 60 ml/min (Cockcroft-Gault), 12. Bilirubin > upper limit of normal (ULN) x 1.2,  In case of suspected Gilbert’s disease: non-fasting total bilirubin ≤ ULN x 1.2 and fasting total bilirubin ≤ ULN x 1.5 are acceptable. 13. Alanine aminotransferase (ALT) > ULN x 1.1, 14. Aspartate aminotransferase (AST) > ULN x 1.2, 15. Creatine kinase (CK) not within normal limits (volunteers with CK elevations between ULN and ULN x 3 may be included if troponin T is negative, and 16. Thyroid stimulating hormone (TSH) not within normal limits, 17. A positive human immunodeficiency virus and hepatitis C antibody screen, 18. A positive result in the drug screening test (at SCR, see section 7.2.2) 19. Any intake of HCQ, chloroquine or travel to malaria risk regions within the last 3 months, 20. Use of any medication (prescription medication, non-prescription medication including multivitamin or herbal preparations) with active ingredients except hormonal contraception and thyroid hormones, or any intake of substances known to induce or inhibit HCQ-metabolizing enzymes or drug transporters within a period of less than 5 times the respective elimination half-life (t_1/2_) with regard to the expected date of the first dose of IMP, 21. Consumption of citrus fruits or products of these fruits within 7 d prior to the expected date of first dose of IMP and expected nonadherence to refrain from such products until V5, 22. Expected nonadherence to refrain from alcohol 24 h prior to V1 until V5 of this trial, or excessive alcohol consumption. 23. Intake of quinine, or consumption of quinine-containing drinks (bitter lemon, tonic water, bitter orange) 24. Use of an IMP within 30 d prior to the expected date of receiving the first dose of IMP or active enrolment in another drug or vaccine clinical trial. 25. Contraindications to HCQ use: 26. Hypersensitivity to the active substance, 4-aminoquinoline, or to any of the excipients 27. History of retinopathy or maculopathy 28. Glucose-6-phosphat-dehydrogenase deficiency (fauvism, haemolytic anemia) 29. Diseases of the haematopoietic system 30. Myasthenia gravis 31. During pregnancy (except for treatment of malaria) 32. While breastfeeding   **At V1, prior to dosing:**   1. Use of any medication (prescription medication, non-prescription medication including multivitamin or herbal preparations) with active ingredients except hormonal contraception and thyroid hormones or any intake of substances known to induce or inhibit HCQ-metabolizing enzymes or drug transporters within a period of less than 5 times the respective elimination half-life (t1/2) with regard to the expected date of the first dose of IMP, and 2. Use of an IMP within 30 d prior to receiving the first dose of IMP or active enrolment in another drug or vaccine clinical trial. |
| Interventions | 11a | Participants are randomized in two groups to either receive a nine-day course of pantoprazole, or to a control group without pantoprazole. All participants receive a single dose of HCQ 400 mg.  Additionally, CYP3A4 and CYP2D6 phenotyping with microdosed probe drugs is performed using midazolam and yohimbine, respectively. |
|  | 11b | Any participant can withdraw from treatment at any time without personal disadvantages and without having to give a reason. Participants who discontinue exposure to the trial treatment on their own will be defined as premature withdrawals. Premature withdrawals will be replaced. The time of withdrawal from trial and/or treatment discontinuation must be documented in the source data and on the CRF.  The investigator can also discontinue the treatment after considering the risk-to-benefit ratio, e.g. if he/she considers that further treatment of the participant according to the trial protocol is no longer justifiable. The date of and the primary reason for the withdrawal from treatment, as well as the observations available at the time of withdrawal will be documented in the CRF.  Reasons leading to the withdrawal of a participant can include but are not limited to the following: intolerable AE, lack of participant’s cooperation, new occurrence of a disease precluding continuation, emergence of major exclusion criteria (coming to light after inclusion) that potentially endanger the safety of the participant.  If consent is not withdrawn, every effort must be made to obtain safety parameters until the EOT visit. |
|  | 11c | - HCQ will be administered by the trial team; therefore, taking and timing adherence of 100 % is expected. A drug accountability log will document the use of drug. - On visits 1 and 2, pantoprazole will be administered by the investigator. On all other days, the participants are responsible for correct administration; intake will be documented in a diary. A drug accountability log will document the use of drug throughout the study. - Administration of midazolam and yohimbine is carried out at the trial site; therefore, a taking and timing adherence of 100 % is expected. A drug accountability log will document the use of drug. |
|  | 11d | Use of any medication (prescription medication, non-prescription medication including multivitamin or herbal preparations) with active ingredients except hormonal contraception and thyroid hormones, or any intake of substances known to induce or inhibit HCQ-metabolizing enzymes or drug transporters within a period of less than 5 times the respective elimination half-life (t1/2) with regard to the expected date of the first dose of IMP. |
| Outcomes | 12 | Primary endpoint:  AUC_0-72 h_ and C_max_ of a single oral dose of 400 mg HCQ with and without pantoprazole.  Secondary endpoints:   - AUC_2-4 h_, AUC_0-6 h_, and Cmax of microdosed midazolam and microdosed yohimbine, - Correlation of concentrations of HCQ in whole blood with concentrations in plasma and PBMCs.   Exploratory endpoints:  AUC_0-72 h_ and C_max_ of major metabolites of HCQ; this may include but is not limited to desethyl-HCQ with and without pantoprazole.  Outcome measures:  Concentration-time curve of HCQ and derived PK parameters in whole blood,  Concentration-time curve of midazolam and yohimbine in plasma,  Concentrations of HCQ and desethyl-HCQ in plasma and PBMCs,  Concentration-time curve of HCQ metabolites and derived PK parameters. |
| Participant timeline | 13 | The trial consists of a screening visit (SCR), a treatment period (s. table 1 and figure 1), and an end-of-trial visit (EOT).  **Treatment period:**  Table 1   | Visit 1 (day 1): | - Breakfast (3 h before drug administration) - Administration of 30 µg midazolam per os (p.o.), and - Administration of 50 µg yohimbine p.o. - Start 0-6 h pharmacokinetic assessments (PK) - After PK assessments start with pantoprazole (p.o.) for 9 d   Timing will be aligned with the expected timing of breakfast and dosing on V2. | | --- | --- | | Visit 2 (day 6): | - PPI therapy continued (morning dose 1 h before breakfast) - 400 mg HCQ p.o. with food - Start 0-24 h HCQ PK - 3 h after HCQ: administration of 30 µg midazolam and 50 µg yohimbine p.o. - Start 0-6 h midazolam and yohimbine PK | | Visit 3 – 5  (day 7-9): | - PPI therapy continued until day 9 - PK sampling 24, 36, 48, and 72 h after HCQ administration | | Figure 1 | | |
| Sample size | 14 | The planned sample size is 24 healthy volunteers, 12 in each arm.  The sample size calculation is based on previous data after single doses of HCQ. An online calculator was used for the sample size calculation (http://hedwig.mgh.harvard.edu/sample_size/js/js_parallel_quant.html). With the AUC_0-72 h_ data available, a sample size of 14 (7 in each group) is needed to detect a 30 % difference in AUC_0-72 h_ with a power of 0.8 and an α-error of 5 %. Therefore, a sample size of 12 per group is regarded as sufficient to detect a PPI-induced difference on HCQ AUC_0-72 h_ exceeding the bioequivalence range (80-125 %).  Up to 12 volunteers may be replaced if enrolled volunteers drop out prior to exposure with HCQ or prior to completing the 72 h PK sampling period. |
| Recruitment | 15 | Up to 12 volunteers may be replaced if enrolled volunteers drop out prior to exposure with HCQ or prior to completing the 72 h PK sampling period. |
| **Methods: Assignment of interventions (for controlled trials)** | | |
| Allocation: |  |  |
| Sequence generation | 16a | A computer-generated randomization list was created. Participants are assigned random numbers based on consecutive enrolment. |
| Allocation concealment mechanism | 16b | Participants are assigned random numbers based on consecutive enrolment. |
| Implementation | 16c | Generation of allocation sequence, enrolment of participants, and assignment of participants to interventions is performed IN the Clinical Pharmacological Trial Center of Heidelberg University Hospital, Germany. |
| Blinding (masking) | 17a | Not applicable |
|  | 17b | Not applicable |
| **Methods: Data collection, management, and analysis** | | |
| Data collection methods | 18a | All data required by the protocol will be recorded on source data.  Participant medical records must be kept for all volunteers taking part in the trial. Participation in the trial, dates of participant information and signed informed consent, administration of trial medication, and key data recorded during the trial must be documented in the participant’s medical record as source data unless specified otherwise in this trial protocol. Data in the participant’s medical record (including original laboratory reports etc.) are the ‘source data’ and entries in the CRF have to be checked against these data (source data verification) by the monitor. |
|  | 18b | Outcome data is collected for all participants who received treatment. Safety data is collected for all participants, including those who discontinue or deviate from intervention protocols. Due to the short duration of the study, participant completion of follow-up is expected to be very likely. |
| Data management | 19 | The data will timely be entered in a database via an electronic CRF (eCRF), according to the trial site’s SOP. The software ensures that the participants´ pseudonymity will be maintained. All data to be recorded according to this trial protocol must be documented in the eCRF. After checking for plausibility, consistency, and completeness, queries will be edited and resolved. Changes in the eCRF are traceable by an audit trail.  After all data are entered into the database and the queries are closed the investigator and deputy will agree on the database closure. |
| Statistical methods | 20a | The primary outcome measures are the difference in AUC_0-72 h_ and C_max_ of a single dose of 400 mg oral HCQ with or without concomitant pantoprazole therapy.  The following parameters will be reported through detailed descriptive statistics and graphics and, if applicable, changes will be analysed using ANOVA or paired t-tests at an α = 5 % level:   - AUC_2-4 h_, AUC_0-6 h_ and C_max_ of microdosed midazolam and microdosed yohimbine, - Correlation of concentrations of HCQ in whole blood as compared to concentrations in plasma and PBMCs. - AUC_0-72 h_ and C_max_ of desethyl-HCQ with and without pantoprazole. - Metabolic rations will be analysed where metabolites are available - Seriousness, severity, relationship, and frequency of AEs during the treatment period will be tabulated and summarized. |
|  | 20b | Not applicable. |
|  | 20c | All volunteers who received trial treatment will be analysed. Analysis of the primary endpoint will be done in the complete case set. Safety analyses will be done with all volunteers who received trial medication.  Individual missing or inconsistent data will be subject to a simple query edit process. Eventually missing data will not be imputed. |
| **Methods: Monitoring** | | |
| Data monitoring | 21a | Qualified personnel from the Department of Clinical Pharmacology and Pharmacoepidemiology will carry out the clinical monitoring. The monitor is responsible for checking the quality of data and adherence to the trial protocol and to legal and ethical requirements according to local laws and the principles of GCP. Source data verification is an essential part of the monitoring process and the investigator must grant direct access to all data.  The monitor will work according to the pertinent versions of the monitoring SOPs of the Department of Clinical Pharmacology and Pharmacoepidemiology.  The following variables will be verified to an extent that will be defined in a monitoring plan prior to trial start:   - Informed consent, - Inclusion/exclusion criteria, - Demographic data (identity number, age, and sex), - Drug accountability, handling, and storage, - AEs and especially SAEs, - Premature trial termination for individual participants (dropouts and withdrawals), and - Adherence to the protocol and to GCP standards. |
|  | 21b | If new information on the risk-to-benefit ratio of the drug or on the treatment methods used in the trial is coming to light and safety concerns arise, the sponsor reserves the right to interrupt or terminate the project. In addition the principal investigator (PI) may decide at any time to terminate the trial due to a situation where an unexpected high frequency of AEs will occur or due to financial reasons.  The study must be stopped if more than one serious or grade IV related AE will have occurred. SAEs definitely caused by accidental injuries will not be counted towards the stopping criteria. Laboratory abnormalities without any clinical symptoms will not count as stopping criteria. |
| Harms | 22 | Any AE must be documented and suspected unexpected serious adverse reactions (SUSAR) will be reported to the responsible authorities (for definition see Appendix 16.1) such as the EC, the Federal Institute for Drugs and Medical Devices (BfArM), and the Eudra Vigilance Database. In all cases, the EU-report form must show the identity of the investigator and be dated and signed. The description of the AE will include the time of onset, duration, seriousness, intensity, outcome, relationship to the study drug and any treatment required. The investigator will assess the intensity and causality of any AE (for classification and assessment see Appendix 1).  In this trial, the overnight stay at V2 does not qualify as SAE.  The observation period begins with the first administration of the IMP (any AEs prior to the first administration of the IMP are documented as medical history) and ends with the EOT visit. The investigator follows the outcome of AEs or abnormal laboratory findings that are related to study activities until recovery or stabilisation of the participant’s state. Further therapeutic consequences will be documented, for example any medical treatment or drop-out of the study.  In the case of a SAE, the Safety Officer of the Department will be informed within 24 h. Notification on a SAE form can be handed over in person, sent by mail or fax, and will be documented.  The initial report must be as complete as possible including details of the current illness and (serious) AE and an assessment of the causal relationship between the event and the trial medication. (The investigator must also inform the site monitor in all cases). |
| Auditing | 23 | The investigator agrees to allow the auditors/inspectors/monitors to have direct access to the trial records for review, being understood that this personnel is bound by professional secrecy, and as such will not disclose any personal identity or personal medical information. The investigator will make every effort to help with the performance of the audits and inspections, giving access to all necessary facilities, data, and documents. |
| Ethics and dissemination | | |
| Research ethics approval | 24 | The investigator submits the required documents to the responsible Ethics Committee (EC) of the Medical Faculty of Heidelberg University, Germany, and obtains the opinion of the Committee in writing. The investigator also submits the required documents to the Bundesinstitut für Arzneimittel und Medizinprodukte (German Federal Institute for Drugs and Medical Devices) (BfArM) and obtains the approval in writing. Participants will not be included until unconditional approval of BfArM and EC has been received. |
| Protocol amendments | 25 | If the trial protocol has to be changed substantially after approval, a written amendment is required that must be signed by the same persons as mentioned in the trial protocol. Any protocol amendment will only be implemented after approval has been granted by the EC and the competent authorities. Any substantial protocol amendment affecting the benefit-to-risk ratio must be approved by the responsible EC and BfArM and must be notified to the local regulatory authority (Regierungspräsidium Karlsruhe). These procedures must be completed before any modifications can come into operation, except when they are necessary to eliminate immediate hazards for the trial participants. Participants will be informed about relevant changes in the trial and will be asked to re-consent in writing |
| Consent or assent | 26a | Before being admitted to the clinical trial, the participant must consent to participate after being fully informed by the investigator or a designated member of the investigating team about the nature, importance, risks and individual consequences of the clinical trial and their right to terminate the participation at any time. |
|  | 26b | Not applicable |
| Confidentiality | 27 | The data obtained in the course of the trial will be treated pursuant to the State Data Protection Law (Landesdatenschutzgesetz Baden Württemberg, Germany) and the General Data Protection Regulation of the EU (DSGVO, EU 2016/679).  During the clinical trial, participants’ identification and results will be documented in a file comparable to a patient file that follows all rules of confidentiality of the Heidelberg University Hospital, Germany.  Relevant data for the trial will be documented in a database in a pseudonymized way in order to prevent any possibility for third parties to identify the participant. Also if data are transferred to third parties or published, they will only be used in the pseudonymized way. Trial findings stored on a computer will be stored in accordance with the data protection law and will be handled in strictest confidence. For protection of this data, organisational procedures are implemented to prevent distribution of data to unauthorised persons. The appropriate regulations of local data will be fulfilled in their entirety.  The participant consents in writing to release the investigator from his professional discretion in so far as to allow inspection of original data for monitoring purposes by health authorities and authorised persons. Authorised persons (clinical monitors, inspectors, and auditors) may take insight into personal participant-related data collected during the trial ensuring the data protection law.  The investigator will maintain a participant identification list (participant numbers with the corresponding names) to enable records to be identified.  Volunteers who did not consent to circulate their pseudonymised data will not be included into the trial. |
| Declaration of interests | 28 | There are no financial and other competing interests for the principal investigator or deputy investigators. |
| Access to data | 29 | All investigators will have access to the final trial dataset. |
| Ancillary and post-trial care | 30 | The sponsor has to subscribe to an insurance policy covering, in its terms and provisions, its legal liability for injuries caused to participating persons and arising out of this research performed strictly in accordance with the scientific protocol as well as with applicable law and professional standards. The insurance also includes the coverage of travel accidents (travel accident insurance). |
| Dissemination policy | 31a | Trials results will be communicated in a peer-reviewed journal. There are no publication restrictions. |
|  | 31b | Authorship eligibility is based on the following criteria (ICMJE):   - Substantial contributions to the conception or design of the work; or the acquisition, analysis, or interpretation of data for the work; AND - Drafting the work or revising it critically for important intellectual content; AND - Final approval of the version to be published; AND - Agreement to be accountable for all aspects of the work in ensuring that questions related to the accuracy or integrity of any part of the work are appropriately investigated and resolved.   There is no intended use of professional writers. |
|  | 31c | Not applicable. |
| Appendices |  |  |
| Informed consent materials | 32 | Not applicable as available only in German language. |
| Biological specimens | 33 | Not applicable. |

The checklist has been created in conjunction with the SPIRIT 2013 Explanation & Elaboration for important clarification on the items.
